# Safety of azithromycin in pediatrics: a systematic review and meta-analysis

**DOI:** 10.1007/s00228-020-02956-3

**Published:** 2020-07-17

**Authors:** Linan Zeng, Peipei Xu, Imti Choonara, Zhenyan Bo, Xiangchen Pan, Wenyan Li, Xiaofeng Ni, Tao Xiong, Can Chen, Leshan Huang, Shamim Ahmad Qazi, Dezhi Mu, Lingli Zhang

**Affiliations:** 1 Department of Pharmacy/Evidence-based Pharmacy Center, West China Second University Hospital, Sichuan University, Chengdu, 610041 China; 2 Key Laboratory of Birth Defects and Related Diseases of Women and Children (Sichuan University), Ministry of Education, Chengdu, 610041 China; 3 West China School of Medicine, Sichuan University, Chengdu, 610041 China; 4 West China School of Pharmacy, Sichuan University, Chengdu, 610041 China; 5 Academic Division of Child Health, University of Nottingham, Derbyshire Children’s Hospital, Derby, DE22 3NE UK; 6 Department of Pediatrics, West China Second University Hospital, Sichuan University, Chengdu, 610041 China; 7 Department of Pharmacy, Zhongshan Hospital, Fudan University, Shanghai, 200032 China; 8 Department of Pharmacy, The Third Affiliated Hospital of Guangzhou Medical University, Guangzhou, 510150 China; 9 Department of Maternal Newborn Child and Adolescent Health, World Health Organization, 20 Avenue Appia, 1211 Geneva, Switzerland

**Keywords:** Azithromycin, Safety, Pediatrics, Systematic review, Meta-analysis

## Abstract

**Purpose:**

To evaluate the toxicity of azithromycin in neonates, infants, and children.

**Methods:**

A systematic review was performed for relevant studies using Medline (Ovid), PubMed, Cochrane Central Register of Controlled Trials, EMBASE, CINAHL, and International Pharmaceutical Abstracts. We calculated the pooled incidence of adverse drug reactions (ADRs) associated with azithromycin based on prospective studies (RCTs and prospective cohort studies) and analyzed the risk difference (RD) of ADRs between azithromycin and placebo or other antibiotics using meta-analysis of RCTs.

**Results:**

We included 133 studies with 4243 ADRs reported in 197,675 neonates, infants, and children who received azithromycin. The safety of azithromycin as MDA in pediatrics was poorly monitored. The main ADRs were diarrhea and vomiting. In prospective non-MDA studies, the most common toxicity was gastrointestinal ADRs (938/1967; 47.7%). The most serious toxicities were cardiac (prolonged QT or irregular heart beat) and idiopathic hypertrophic pyloric stenosis (IHPS). Compared with placebo, azithromycin did not show increased risk ADRs based on RCTs (risk difference − 0.17 to 0.07). The incidence of QT prolonged was higher in the medium-dosage group (10–30 mg/kg/day) than that of low-dosage group (≤ 10 mg/kg/day) (82.0% vs 1.2%).

**Conclusion:**

The safety of azithromycin as MDA needs further evaluation. The most common ADRs are diarrhea and vomiting. The risk of the most serious uncommon ADRs (cardiac-prolonged QT and IHPS) is unknown.

**Electronic supplementary material:**

The online version of this article (10.1007/s00228-020-02956-3) contains supplementary material, which is available to authorized users.

## Introduction

Azithromycin is an acid-stable orally administered macrolide antimicrobial drug, structurally related to erythromycin [1]. Due to its broad antibacterial spectrum against *Streptococcus pneumonia*, *Moraxella catarrhalis*, and atypical pathogens, azithromycin has been used extensively for the treatment of pediatric infectious diseases and became one of the most commonly prescribed antibiotics in children [2–6]. During the last 20 years, azithromycin mass drug administration (MDA) has been used to control trachoma with over 700 million doses of azithromycin being prescribed to children in areas of active trachoma programs [7]. Recent large trials have suggested that periodical azithromycin MDA may reduce post-neonatal infant and child mortality [8]. However, the long-term rationale for mass antibiotic distribution for trachoma is still the subject of debate with concerns of potential toxicity with azithromycin in pediatrics [9, 10].

A systematic review that evaluated the tolerance or toxicity of azithromycin in children with asthma found that gastrointestinal adverse reactions such as nausea, diarrhea, and abdominal pain were the main adverse events [11]. Another systematic review of azithromycin use in neonates highlighted the risk of infantile hypertrophic pyloric stenosis (IHPS) [12]. This systematic review aims to evaluate the toxicity of azithromycin both as MDA or non-MDA in neonates, infants, and children from birth to 18 years old. This systematic review was proposed by the World Health Organization (WHO), as one of the systematic reviews in support of developing a guideline of azithromycin use in pediatrics to help national and international policymakers in determining the role of prophylactic azithromycin in reducing child mortality [10].

## Methods

This systematic review conformed to the PRISMA statement and was registered on PROSPERO (CRD 42018112629) [13]. We have reported the methods of literature search, risk of bias assessment, data abstraction, and data analysis in the published protocol [14].

### Search strategy and literature search

In brief, we performed a comprehensive search using Medline (Ovid), PubMed, Cochrane Central Register of Controlled Trials, EMBASE, CINAHL, and International Pharmaceutical Abstracts. We reported the search strategies for each database in the published protocol [14]. We included randomized controlled trials (RCTs), cohort studies, case–control studies, cross-sectional studies, case series, and case reports that included pediatric patients (aged from birth to 18 years old) using azithromycin as periodic MDA or as therapeutic agent for any disease till March 2019, and updated the search in September 2019. We had no restriction on language. We excluded editorials, conference abstracts, and reviews. We searched for adverse drug reactions (ADRs) reported in spontaneous reporting systems or safety communication announcements as planned. However, since none of the spontaneous reporting systems provided detailed information for individual ADRs (e.g., age of patient, dosage of azithromycin) and none of the announcements was based on evidence in patients younger than 16 years old, we did not present the result in this paper.

### Data extraction and analysis

We abstracted study design, characteristic of patients, interventions, methods used for safety monitoring, and ADRs from the eligible studies. We used the Cochrane risk of bias tool to assess risk of bias in RCTs, the Newcastle-Ottawa Scale for case–control study and cohort studies, and the Joanna Briggs Institute (JBI) Critical Appraisal tools for case series, case reports, and analytical cross-sectional studies [15–17]. We used the Grading of Recommendations Assessment, Development and Evaluation (GRADE) to assess the certainty of body of evidence [18]. We categorized ADRs by systems according to the Medical Dictionary for Regulatory Activities (MedDRA) Terminology (version 21.1) [19] and by frequencies according to the Council for International Organization of Medical Science (CIOMS) as very common (≥ 10%), common (≥ 1% and < 10%), uncommon (≥ 0.1% and < 1%), and rare (< 0.1%) [20]. When the primary study explicitly reported zero ADR or when the study had the capacity to detect the ADR but did not report any event, we counted it as a zero event [21].

We calculated the pooled incidence of ADRs associated with azithromycin based on RCTs and prospective cohort studies to determine the risk of individual ADRs [22, 23]. We categorized ADRs into those that needed specific investigations (e.g., “decreased white blood cells” needs to be detected by a blood test; “prolonged QT” needs to be detected by an electrocardiograph (ECG)) and those that could be observed without specific investigations (e.g., diarrhea, vomiting). We analyzed risk difference (RD) of ADRs between azithromycin and placebo or other antibiotics using meta-analysis of RCTs. We did not use relative risk (RR) or odds ratio (OR) as planned in the protocol, as one cannot use inverse-variance methods to calculate RR or OR, when the number of events is zero in either group. One can, however, still calculate RD [24].

We used chi-square test of contingency table to identify the difference of pooled incidence of ADRs between different dosage groups (≤ 10 mg/kg/day, 10–30 mg/kg/day, > 30 mg/kg/day). The number of studies in each category did not meet the criteria for conducting regression (at least 10 events per category of dependent variables and 10 events per category of independent variables).

## Results

**Fig. 1:**
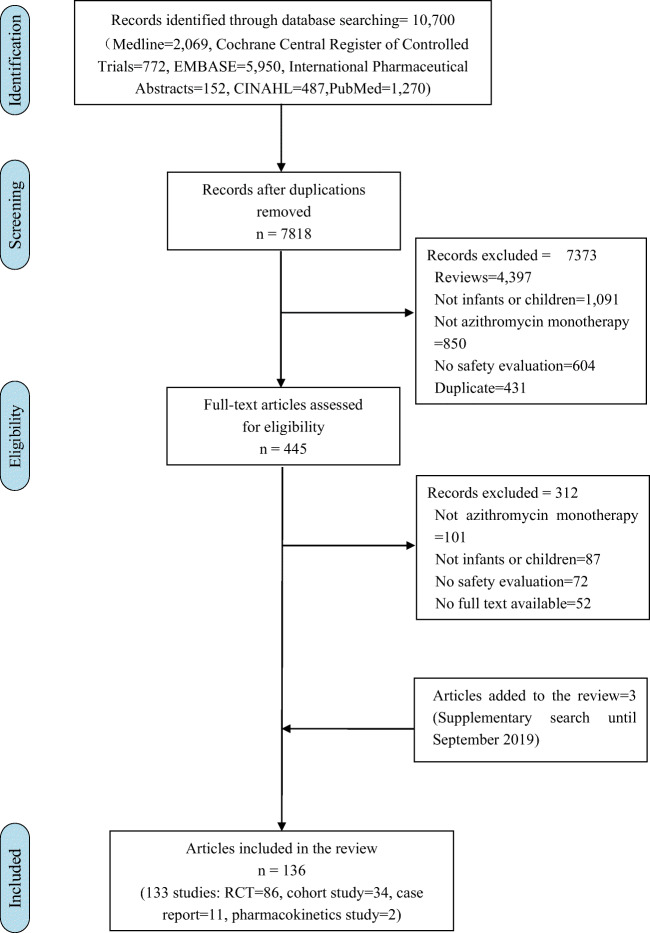
Flowchart for screened articles

Of 10,700 titles identified, 445 proved potentially eligible after reviewing abstracts for the systematic reviews; 131 studies (133 articles) proved eligible following full text review. The updated search until September 2019 found two new trials (three articles) (Fig. 1). ESM Appendix 1 presents the characteristics of eligible studies. Risk of bias of individual RCTs was mainly due to unblinding of participants (high risk or unclear 65.1%, 56/86) or unblinding of outcome assessment (high risk or unclear 70.9%, 61/86). Risk of bias of individual cohort studies was mainly due to the lack of demonstration that outcome of interest was not present at the start of the study (55.9%, 19/34) and the lack of comparability of cohorts (76.5%, 26/34) (ESM Appendix 2). Among all types of studies, 4243 ADRs were reported from 197,675 pediatric patients who received azithromycin. The majority of ADRs (56.1%, 2382/4243) were reported from RCTs not as MDA, 14.2% (603/4261) from retrospective cohort studies, and 6.5% (274/4243) from prospective cohort studies (Table 1).

**Table 1:** Summary of all articles

Study type	Number of studies	Number of ADRs (%)	Number of patients (%)
RCT not as MDA	77	2382 (56.1)	9830 (5.0)
Studies of MDA	10*	948 (22.3)	154,180 (78.0)
Prospective cohort	25	274 (6.5)	2374 (1.2)
Retrospective cohort	8**	603 (14.2)	31,209 (15.8)
Case report	11	13 (0.3)	12 (0.01)
Pharmacokinetics	2	23 (0.5)	70 (0.04)
Total	133	4243	197,675

*ADR*, adverse drug reaction; *MDA*, mass drug distribution; *RCT*, randomized controlled trials

*Nine RCTs and one prospective cohort study

**One retrospective cohort study (*n* = 5039) focused on cardiac arrest in pediatric patients receiving azithromycin, and another retrospective cohort study (*n* = 15,073) focused on tendon or joint disorders

### Azithromycin as MDA

We included eight RCTs and one prospective cohort study for azithromycin as MDA. Five RCTs reported no serious ADRs [25–29]. The incidence of ADRs was slightly higher in mass oral azithromycin communities compared with the untreated communities, and the most common ADRs were abdominal pain and vomiting in surveillance for adverse events during a large RCT [30]. The MORDOR study reported 11 cases of serious ADRs including 4 malaria, 1 respiratory infection, 1 ileus, 1 coma, and 4 deaths among 97,047 children through spontaneous reporting system reported by village informants and health facilities [31]. A cluster RCT of biannual mass azithromycin in Niger among preschool children found that the most common guardian-reported ADRs were diarrhea (110/571 in the azithromycin group, 321/1141 in the placebo group, *P* = 0.03), vomiting (91/571 in the azithromycin group, 240/1141 in the placebo group, *P* = 0.07), and rash (70/571 in the azithromycin group, 155/1141 in the placebo group, *P* = 0.07). This study found no statistically significant difference in the incidence of ADRs between azithromycin as MDA and placebo (RR 0.86, 95% CI 0.68 to 1.10, *P* = 0.23) [32, 33]. A pilot cohort study in Ghana reported that 45 out of 14,548 children (0.3%) in azithromycin as MDA had mild to moderate self-limiting ADRs including abdominal discomfort, nausea, and vomiting reported by trained volunteers [34].

### Azithromycin not as MDA

#### Prospective studies

For azithromycin administered not as MDA, a total of 1967 ADRs were reported in 10,132 patients from 63 RCTs and 22 prospective cohort studies. Gastrointestinal (GI) ADRs were most common, accounting for almost half of the ADRs (938/1967, 47.7%), followed by abnormal investigations (239/1967, 12.2%) and respiratory, thoracic, and mediastinal ADRs (186/1967, 9.4%) (Tables 2 and 3). Diarrhea was the most common event among GI ADRs, accounting for 18.3% of all ADRs with a risk of 3.56 per 100 patients. Other common ADRs included vomiting (2.56 per 100 patients), abdominal pain (1.37 per 100 patients), and nausea (0.71 per 100 patients) (Tables 2 and 3).

**Table 2:** Risk of ADRs of azithromycin not as MDA from RCTs and prospective cohort studies (total number of participants = 10,132)

ADRs	No. of events	Pooled incidence of ADRs per 100 participants
Gastrointestinal disorders		
Diarrhea	361	3.56
Vomiting	259	2.56
Abdominal pain	139	1.37
Nausea	72	0.71
Loose stools	69	0.68
Abdominal pain upper	19	0.19
Flatulence	6	0.06
Stomachache	6	0.06
Gastrointestinal adverse event	7	0.07
Subtotal	938	
Respiratory, thoracic, and mediastinal disorders		
Cough	75	0.74
Nasal congestion	46	0.45
Pharyngolaryngeal pain	29	0.29
Rhinorrhoea	25	0.25
Cough productive	11	0.11
Subtotal	186	
General disorders and administration site conditions		
Fever	165	1.63
Fatigue	10	0.10
Subtotal	175	
Skin and subcutaneous tissue disorders		
Rash	111	1.10
Hives	10	0.10
Dermatitis	8	0.08
Fungal dermatitis	5	0.05
Subtotal	134	
Nervous system disorders		
Headache	49	0.48
Dizziness	7	0.07
Somnolence	6	0.06
Subtotal	62	
Metabolism and nutrition disorders		
Anorexia	22	0.22
Decreased appetite	10	0.10
Subtotal	32	
Immune system disorders		
Jarisch–Herxheimer’s reaction	13	0.13
Subtotal	13	
Miscellaneous^*^	102	1.01
Total	1642	

We excluded 14 RCTs and 3 prospective cohorts from the calculation of pooled incidences of ADRs due to the lack of detailed description of ADRs

*ADR*, adverse drug reaction; *MDA*, mass drug administrations

*ADRs with pooled incidence less than 5 per 100 participants

**Table 3:** Risk of special ADRs of azithromycin not as MDA from RCTs and prospective cohort studies

ARDs	No. of events	No. of studies	No. of participants	Pooled incidence of ARDs per 100 participants
Abnormal investigations				
Increased eosinophils	67	52	5278	1.27
Decreased white blood cells	52	53	5360	0.97
Decreased neutrophils	35	52	5278	0.66
Increased glutamic-pyruvic transaminase	31	50	5044	0.61
Increased aspartate aminotransferase	11	49	5009	0.22
Thrombocytosis	9	50	5120	0.18
Abnormal liver function test	8	51	5034	0.16
Pulmonary function decreased	14	5	448	3.13
Increased white blood cell counts	4	53	5361	0.07
Thrombocytopenia	3	50	5120	0.06
Increased platelet count	3	50	5120	0.06
*Pseudomonas* test positive	2	1	110	1.82
Subtotal	239			
Cardiac disorders				
Electrocardiogram QT prolonged*	54	5	277	19.49
Electrocardiogram QT shortened	11	4	157	7.01
Irregular heart beat	10	2	157	6.37
Elevated heart rate	4	2	157	2.55
Subtotal	79			
Miscellaneous***	7	51	5154	0.14
Total	325			

*ADR*, adverse drug reaction; *MDA*, mass drug administrations; *RCT*, randmised controlled trial

*Including borderline QT

**We excluded 14 RCTs and 3 prospective cohorts from the calculation of pooled incidences of ARDs for the lack of detailed description of ADRs

***ADRs that only has one event reported

**Fig. 2:**
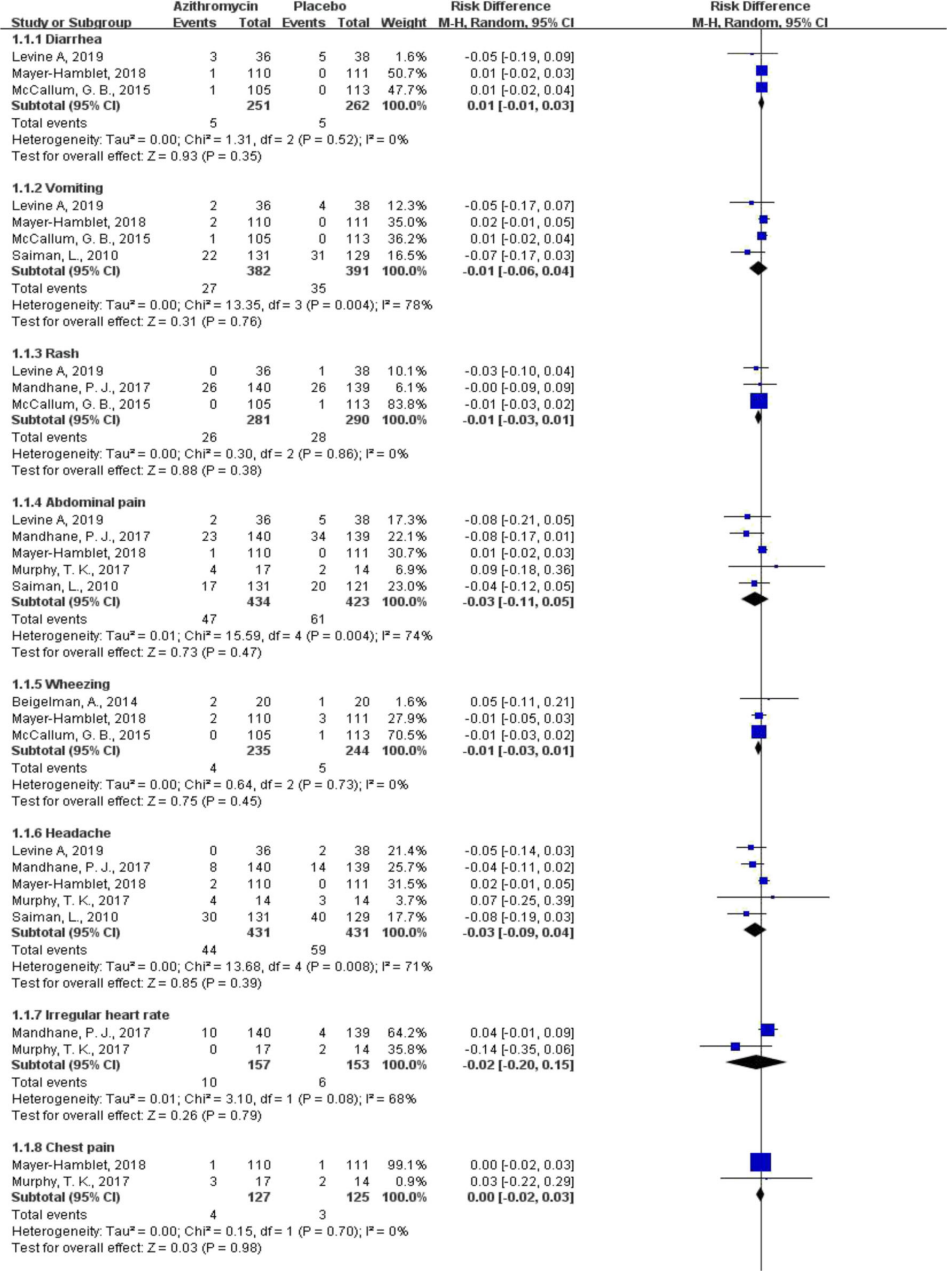

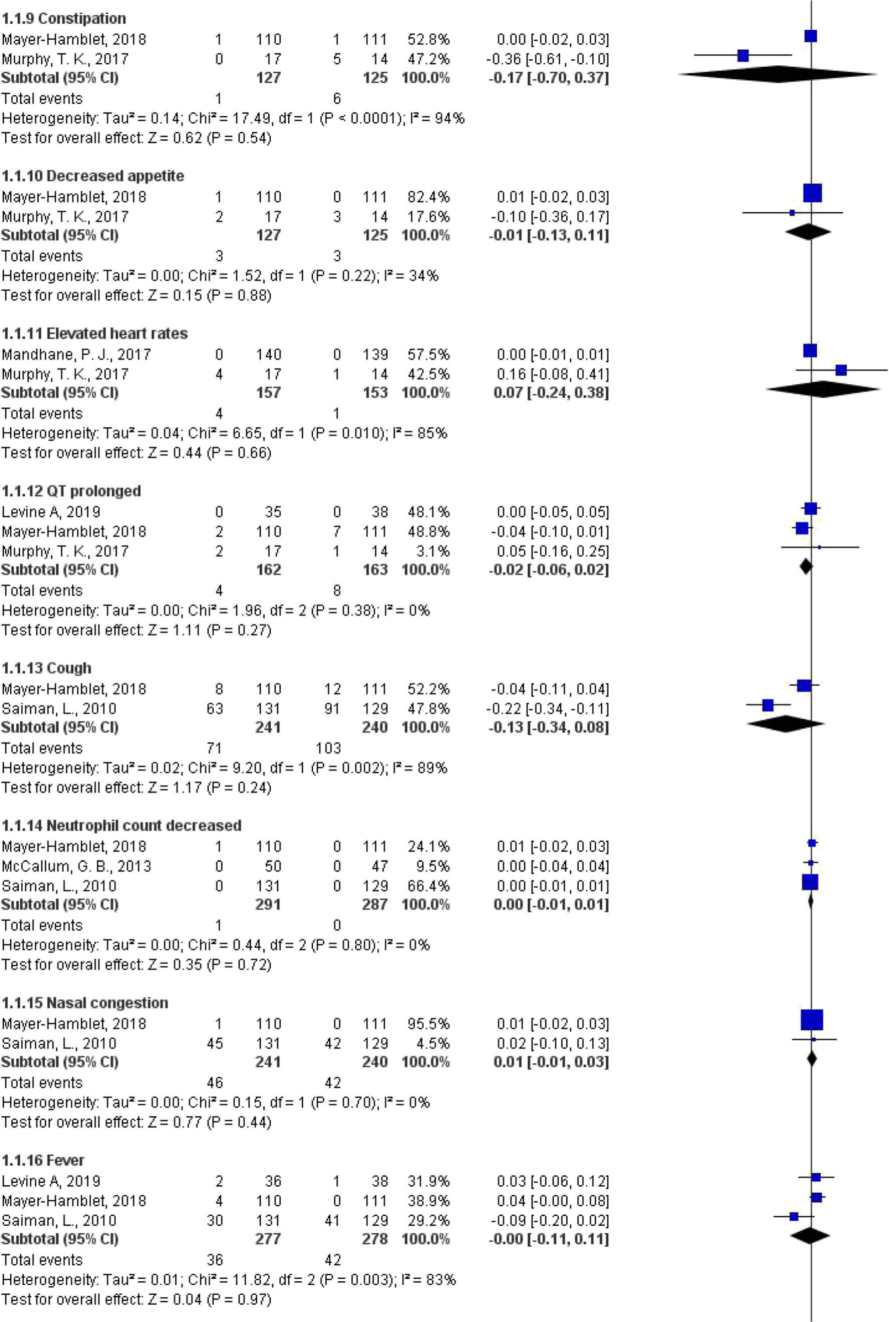

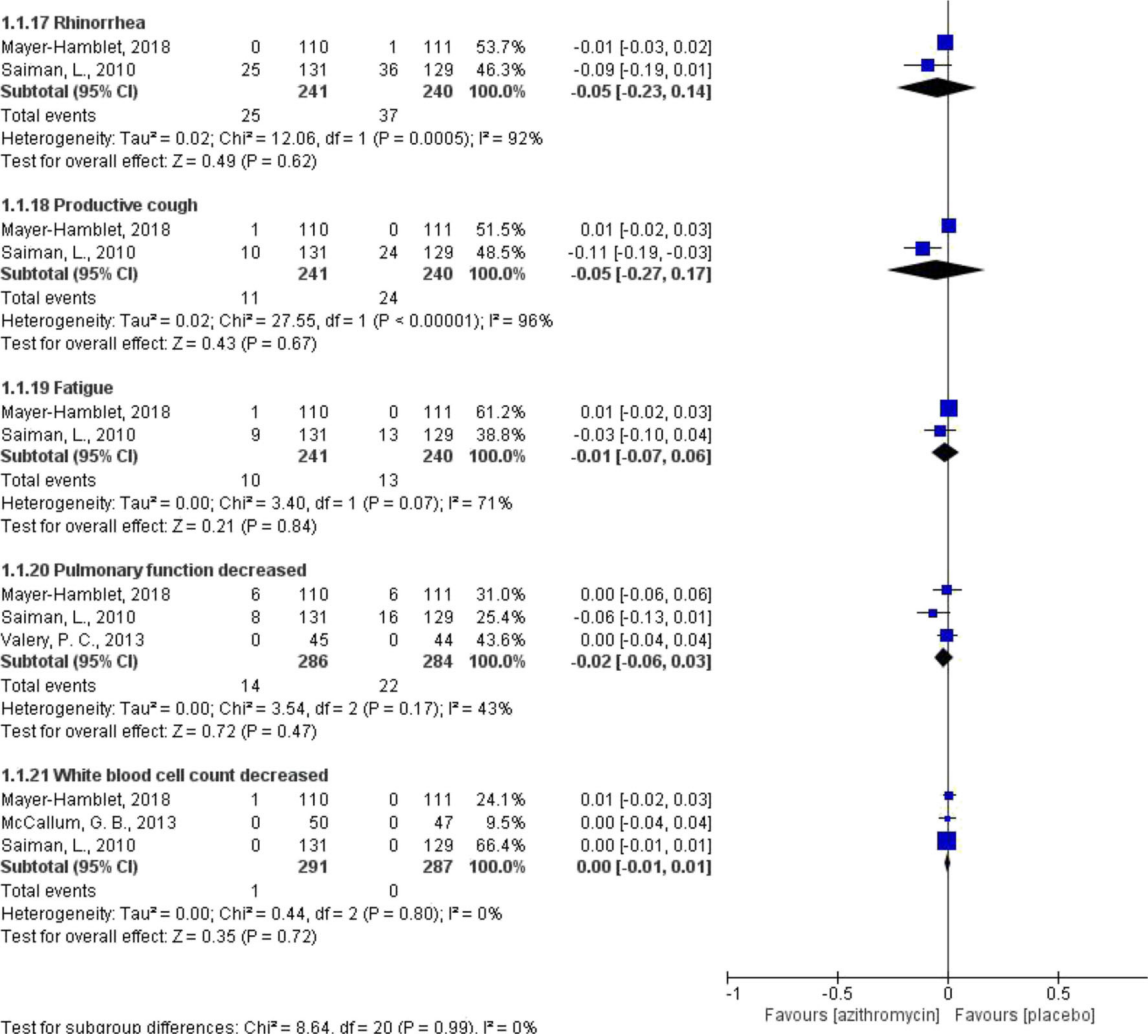
Risk difference of ADRs between azithromycin not as MDA and placebo

Compared with placebo, azithromycin showed no statistically significant difference in terms of incidence of ADRs (risk difference [RD] − 0.17 to 0.07; low to moderate certainty of evidence) (Fig. 2). Similar results were found when azithromycin was compared with cephalosporins and other macrolides (ESM Appendix 3). Azithromycin showed a decreased risk of diarrhea, when compared with amoxicillin/clavulanate (RD − 0.11, 95% CI − 0.15 to − 0.07, *P* < 0.001; low certainty of evidence), but an increased risk of diarrhea when compared with penicillin V (RD 0.03, 95% CI 0.01 to 0.05, *P* = 0.005; high certainty of evidence) (ESM Appendix 3). ESM Appendix 4 presents certainty of evidence of each pooled estimate.

#### Retrospective studies

Three hundred and six ADRs were reported in 31,221 patients from eight retrospective cohort studies and 11 case report studies (ESM Appendix 1). The ADRs reported in retrospective studies were generally consistent with those in prospective studies. GI ADRs were still the most common events accounting for 44.9% (145/323) of all ADRs, followed by musculoskeletal and connective tissue ADRs (36.5%, 118/323). However, some ADRs that were not detected in prospective studies were reported by retrospective studies including tendon or joint disorders (TJDs) (*n* = 118), infantile IHPS (*n* = 8), and ventricular tachycardias (*n* = 4). The full list is shown in ESM Appendix 4.

### Cardiac toxicity

Most prospective studies in children did not evaluate the risk of cardiac toxicity. Cardiac toxicity was only studied or reported in eight studies (six prospective, one retrospective, and one case report). Among prospective studies, five RCTs and one prospective cohort study reported 79 cardiac adverse events (Table 4) [35–40]. One prospective study where children received weekly azithromycin for 6 months reported statistically significant QT prolongation [35]. QT prolongation was also noted in two other studies. Two studies reported irregular heart rates and two studies reported no adverse events. A retrospective cohort study that compared azithromycin (*n* = 5039) with penicillin or cephalosporin (*n* = 77,943) in pediatric patients found that the rate of cardiac arrest in the azithromycin group was lower than that of the penicillin or cephalosporin groups (0.04% vs 0.14%, *P* = 0.04) [41]. A case report described severe bradyarrhythmia in a 9-month-old infant who received over 50 mg/kg azithromycin intravenously over 20 min [42].

**Table 4:** RCTs and prospective cohort studies that monitored cardiac ADRs

Study ID	Study design	Cardiac ADRs	Incidence in the AZ group	Incidence in the control group	Who monitored the cardiac ADRs	Methods for monitoring cardiac ADRs	Severity of the cardiac ADRs	Duration of follow-up (days)
Murphy T K 2017	RCT	Heart beat irregular or pounding	17.6%, 3/17	Placebo 14.2%, 2/14	Pediatric cardiologists	ECG in the AZ and control groups	Mild, no medication discontinuation	28
		QT borderline	11.8%, 2/17	Placebo 0%, 0/14				
		Elevated heart rate	23.5%, 4/17	Placebo 7.1%, 1/14				
Mandhane P J 2017	RCT	Irregular heart rate	7.1%, 10/140	Placebo 2.9%, 4/139	NR	ECG in the AZ and control groups	No serious or life-threatening adverse events	35
El Hennawi D E D 2017	RCT	QT prolongation	82.0%, 50/61	Benzathine penicillin: not monitored	NR	ECG only in the AZ group	The mean of QT rose significantly from 41.6 + 1.7 ms before treatment to 43.8 + 2.9 ms (*P* = 0.007) after treatment in the AZ group	180
		QT shortening	18.0%, 11/61					
Mayer-Hamblett N 2018	RCT	Prolongation of QTc	1.8%, 2/110	Placebo 6.3%, 7/111	NR	ECG	No clinically significant prolongation of QTc	AZ 11.5 ± 6.1 months Placebo 10.8 ± 6.3 months
Levine A 2019	RCT	No cardiac ADR	0%, 0/35*	Metronidazole 0%, 0/38	Physician	ECG in the AZ and control groups	–	56
Liu S 2018	Prospective cohort**	No cardiac ADR	0%, 0/44	–	Pediatric cardiologists	ECG in the AZ group	–	10

*AZ*, azithromycin; *ADR*, adverse drug reaction; *ECG*, electrocardiograph; *NR*, not reported; *RCT*, randomized controlled trial

*AZ plus metronidazole

**Single-arm cohort

### Pyloric stenosis

A retrospective cohort study utilized a large health system database and evaluated 1,074,236 children born over a period of 12 years [43]; 2466 infants developed IHPS and 4875 infants received azithromycin in the first 90 days of life and eight of these infants (all boys) developed IHPS. The study demonstrated an increased risk following exposure to azithromycin in the first 2 weeks of life (adjusted OR [aOR] of 8.26, 95% CI 2.62–26.0) [43]. Azithromycin exposure between 15 and 42 days also increased the risk of IHPS (aOR of 2.98, 95% CI 1.24–7.20).

### Subgroup analysis of dosage

The incidence of diarrhea, vomiting, fever, and rash was higher in the high-dosage group compared with the low- or medium-dosage group (*P* < 0.01) (Table 5). The incidence of prolonged QT and increased eosinophils was higher in the medium-dosage group than in the low-dosage group (*P* < 0.01) (Table 5).

**Table 5:** Pooled incidence of ADRs from RCTs and prospective cohort studies in different dose groups

ADRs	Low dosage (≤ 10 mg/kg)	Medium dosage (10–30 mg/kg)	High dosage (> 30 mg/kg)	*P* value
ADRs that did not need specific investigations*				
Diarrhea	147 (2.5%)^a^	85 (3.5%)^a^	42 (9.3%)^b^	< 0.001
Vomiting	113 (1.9%)^a^	65 (2.6%)^a^	48 (10.7%)^b^	< 0.001
Abdominal pain	98 (1.7%)^a^	28 (1.1%)^a^	11 (2.4%)^a^	0.059
Fever	9 (0.2%)^a^	0 (0.0%)^a^	7 (1.6%)^b^	< 0.001
Rash	73 (1.3%)^a^	15 (0.6%)^b^	23 (5.1%)^c^	< 0.001
ADRs that needed specific investigation				
QT prolonged^α^	4 (1.2%)^a^	50 (82.0%)^b^	–	< 0.001
Pulmonary function decreased^β^	14 (4.3%)^a^	0 (0.0%)^a^	–	0.10
Increased eosinophil^γ^	38 (0.9%)^a^	31 (3.0%)^b^	–	< 0.001

There was no statistical difference between the two groups with the same letter a, b, or c in the following table. Otherwise, there is a statistical difference

*ADR*, adverse drug reaction; *RCT*, randomized controlled trial

*The total number of patients was 5811 in the low-dosage group, 2454 in the medium-dosage group, and 450 in the high-dosage group

## Discussion

Our review found that the main toxicity of azithromycin in pediatrics was gastrointestinal toxicity, specifically diarrhea, vomiting, and abdominal pain. Based on available data, the main ADRs of azithromycin as MDA were diarrhea and vomiting. However, the reporting of ADRs in RCTs of MDA was very variable. Concerns about the reporting of ADRs in RCTs involving children have been reported by several groups [44, 45]. For azithromycin not as MDA, our review highlighted that the most common toxicity was GI adverse reactions, and the most serious toxicities were cardiac adverse reactions and IHPS. The risk of cardiac toxicity and IHPS following MDA is unknown and can only be determined by prospective surveillance studies. The dose of azithromycin was associated with the risk of ADRs. The higher the dosage, the higher the risk of an ADR.

Our study has several strengths. Using rigorous systematic review methods, we did a comprehensive search of the literature and evaluated the safety of azithromycin as both MDA and not as MDA. Our review included recently published studies which were not included in prior reviews, and thus, summarized all of the available evidence, providing optimal insight into the safety of azithromycin in pediatrics. We assessed the risk of bias of each primary study using risk of bias assessment tools based on study design. We detected the incidence of adverse events based on prospective studies from which the data are more reliable, and explored uncommon events from the larger retrospective studies.

This review also has some limitations. First, the ADRs were poorly reported in primary studies which led to the probable underestimate of the incidence of ADRs in this review. Even the meta-analysis might still be underpowered to detect potential differences between azithromycin and placebo or other antibiotics. The lack of ADR reporting is more common in studies of azithromycin as MDA and we were unable to conclude the safety of azithromycin as MDA compared with placebo. Second, we did not perform analysis of different age groups as planned in the protocol since most primary studies did not report the outcomes for each age group separately.

To our knowledge, the ADRs of azithromycin as MDA in children have not been evaluated in previous systematic reviews [46]. For azithromycin not as MDA, cardiac toxicity of azithromycin has been a concern for a long time. Previous studies and reviews found the risk of azithromycin appears to depend on age and prior cardiovascular risk in adults. However, none of them evaluated the risk in pediatric patients. In our review, cardiac adverse events were found in pediatric patients, but the difference between the azithromycin group and the control group was not significant in most of the studies. Only one study found a statistically significant increase in QT prolongation, but this study involved weekly administration of azithromycin for 6 months and only children who received azithromycin received ECGs [35]. Additionally, the data from some studies must be questioned because the method for safety surveillance was poorly reported [26, 27, 35]. Future studies in pediatric patients should be conscious of using robust methods for safety monitoring.

Previous studies have demonstrated the association between erythromycin exposure early in life and IHPS [47–52]. Only one retrospective study was found in our review that evaluated the association between azithromycin and IHPS. Since azithromycin and erythromycin are slightly different in molecular structure, further studies are required to determine the relationship between early exposure to azithromycin in life and IHPS [12]. The risk of IHPS following MDA is unknown and should be considered in future studies of MDA. However, prospective surveillance following MDA with larger numbers of patients is more likely to be beneficial.

## Conclusion

The main ADRs of azithromycin whether used as MDA or not were gastrointestinal, specifically diarrhea, abdominal pain, and vomiting. For azithromycin not as MDA, the most serious toxicities were cardiac adverse reactions and IHPS. Increasing dose proved to increase the risk of ADRs.

## Electronic supplementary material

ESM 1 (DOCX 22.3 kb)

ESM 2 (DOCX 21 kb)

ESM 3 (PDF 1044 kb)

ESM 4 (DOC 132 kb)

ESM 5 (DOCX 21.1 kb)
