# Supplementary material for: Safety of azithromycin in pediatrics: a systematic review and meta-analysis
Source: Eur J Clin Pharmacol. 2020 Jul 17;76(12):1709–21. doi: 10.1007/s00228-020-02956-3 (PMC7661415; doi:10.1007/s00228-020-02956-3)
Supplement: Supplementary file 1 — (DOCX 22.3 kb) [file 228_2020_2956_MOESM1_ESM.docx]

**Table 5 Pooled incidence of ADRs from RCTs and prospective cohort studies in different dose groups***

| ADRs | Low dosage (≤10mg/kg) | Medium dosage  (10-30mg/kg) | High dosage (>30mg/kg) | P-value |
| --- | --- | --- | --- | --- |
| ADRs that did not need specific investigations ** | | | | |
| Diarrhea | 147(2.5%)^a^ | 85(3.5%)^a^ | 42(9.3%)^b^ | <0.001 |
| Vomiting | 113(1.9%)^a^ | 65(2.6%)^a^ | 48(10.7%)^b^ | <0.001 |
| Abdominal pain | 98(1.7%)^a^ | 28(1.1%)^a^ | 11(2.4%)^a^ | 0.059 |
| Fever | 9(0.2%)^a^ | 0(0.0%)^a^ | 7(1.6%)^b^ | <0.001 |
| Rash | 73(1.3%)^a^ | 15(0.6%)^b^ | 23(5.1%)^c^ | <0.001 |
| ADRs that needed specific investigation | | | | |
| QT prolonged^α^ | 4(1.2%)^a^ | 50(82.0%)^b^ | - | <0.001 |
| Pulmonary function decreased^β^ | 14(4.3%)^a^ | 0(0.0%)^a^ | - | 0.10 |
| Increased eosinophil^γ^ | 38(0.9%)^a^ | 31(3.0%)^b^ | - | <0.001 |

Note: ADR: adverse drug reaction; RCT: randomized controlled trial

*

There was no statistical difference between the two groups with the same letter a, b, or c in the following table. Otherwise, there is a statistical difference.

** The total number of patients was 5,811 in low dosage group, 2,454 in medium dosage group, 450 in high dosage group.
