# Supplementary material for: Safety of azithromycin in pediatrics: a systematic review and meta-analysis
Source: Eur J Clin Pharmacol. 2020 Jul 17;76(12):1709–21. doi: 10.1007/s00228-020-02956-3 (PMC7661415; doi:10.1007/s00228-020-02956-3)
Supplement: Supplementary file 2 — (DOCX 21 kb) [file 228_2020_2956_MOESM2_ESM.docx]

**Table 2 Risk of ADRs of azithromycin not as MDA from RCTs and prospective cohort studies (total number of participants=10,132)***

| **ADRs** | **No. of events** | **Pooled incidence of ADRs per 100 participants** |
| --- | --- | --- |
|  |  |  |
| **Gastrointestinal disorders** |  |  |
| Diarrhea | 361 | 3.56 |
| Vomiting | 259 | 2.56 |
| Abdominal pain | 139 | 1.37 |
| Nausea | 72 | 0.71 |
| Loose stools | 69 | 0.68 |
| Abdominal pain upper | 19 | 0.19 |
| Flatulence | 6 | 0.06 |
| Stomachache | 6 | 0.06 |
| Gastrointestinal adverse event | 7 | 0.07 |
| **Subtotal** | **938** |  |
| **Respiratory, thoracic and mediastinal disorders** |  |  |
| Cough | 75 | 0.74 |
| Nasal congestion | 46 | 0.45 |
| Pharyngolaryngeal pain | 29 | 0.29 |
| Rhinorrhoea | 25 | 0.25 |
| Cough productive | 11 | 0.11 |
| **Subtotal** | **186** |  |
| **General disorders and administration site Conditions** |  |  |
| Fever | 165 | 1.63 |
| Fatigue | 10 | 0.10 |
| **Subtotal** | **175** |  |
| **Skin and subcutaneous tissue disorders** |  |  |
| Rash | 111 | 1.10 |
| Hives | 10 | 0.10 |
| Dermatitis | 8 | 0.08 |
| Fungal Dermatitis | 5 | 0.05 |
| **Subtotal** | **134** |  |
| **Nervous system disorders** |  |  |
| Headache | 49 | 0.48 |
| Dizziness | 7 | 0.07 |
| Somnolence | 6 | 0.06 |
| **Subtotal** | **62** |  |
| **Metabolism and nutrition disorders** |  |  |
| Anorexia | 22 | 0.22 |
| Decreased appetite | 10 | 0.10 |
| **Subtotal** | **32** |  |
| **Immune system disorders** |  |  |
| Jarisch-Herxheimer's reaction | 13 | 0.13 |
| **Subtotal** | **13** |  |
| **Miscellaneous^**^** | 102 | 1.01 |
| **Total** | **1,642** |  |

Note: ADR: Adverse drug reaction; MDA: Mass drug administrations

*We excluded 14 RCTs and 3 prospective cohorts from the calculation of pooled incidences of ADRs due to the lack of detailed description of ADRs.

** ADRs with pooled incidence less than 5 per 100 participants.
