# Supplementary material for: Safety of azithromycin in pediatrics: a systematic review and meta-analysis
Source: Eur J Clin Pharmacol. 2020 Jul 17;76(12):1709–21. doi: 10.1007/s00228-020-02956-3 (PMC7661415; doi:10.1007/s00228-020-02956-3)
Supplement: Supplementary file 3 — (PDF 1044 kb) [file 228_2020_2956_MOESM3_ESM.pdf]

## Appendix 3a: Risk difference of ADRs between azithromycin and penicillin V

| Study or Subgroup | Azithromycin Events | Azithromycin Total | Penicillin V Events | Penicillin V Total | Weight | Risk Difference M-H, Random, 95% CI |
|---|---|---|---|---|---|---|
| **2.1.1 Diarrhea** | | | | | | |
| Hamill, J., 1993 | 1 | 49 | 0 | 47 | 11.6% | 0.02 [-0.03, 0.08] |
| O'Doherty, B. ,1996 | 9 | 326 | 0 | 163 | 88.4% | 0.03 [0.01, 0.05] |
| **Subtotal (95% CI)** | | **375** | | **210** | **100.0%** | **0.03 [0.01, 0.05]** |
| Total events | 10 | | 0 | | | |
| Heterogeneity: $\text{Tau}^2 = 0.00$; $\text{Chi}^2 = 0.06$, df = 1 (P = 0.81); $I^2 = 0\%$ | | | | | | |
| Test for overall effect: Z = 2.78 (P = 0.005) | | | | | | |
| **2.1.2 Vomit** | | | | | | |
| Hamill, J., 1993 | 1 | 49 | 0 | 47 | 17.8% | 0.02 [-0.03, 0.08] |
| O'Doherty, B. ,1996 | 20 | 326 | 2 | 163 | 26.3% | 0.05 [0.02, 0.08] |
| Schaad, U. B, 1996 | 1 | 170 | 0 | 173 | 31.4% | 0.01 [-0.01, 0.02] |
| Schaad, U. B, 2002 | 0 | 141 | 5 | 130 | 24.6% | -0.04 [-0.07, -0.00] |
| **Subtotal (95% CI)** | | **686** | | **513** | **100.0%** | **0.01 [-0.02, 0.04]** |
| Total events | 22 | | 7 | | | |
| Heterogeneity: $\text{Tau}^2 = 0.00$; $\text{Chi}^2 = 14.00$, df = 3 (P = 0.003); $I^2 = 79\%$ | | | | | | |
| Test for overall effect: Z = 0.52 (P = 0.61) | | | | | | |
| **2.1.3 Headache** | | | | | | |
| Hamill, J., 1993 | 1 | 49 | 0 | 47 | 8.5% | 0.02 [-0.03, 0.08] |
| Schaad, U. B, 1996 | 0 | 170 | 1 | 173 | 91.5% | -0.01 [-0.02, 0.01] |
| **Subtotal (95% CI)** | | **219** | | **220** | **100.0%** | **-0.00 [-0.02, 0.01]** |
| Total events | 1 | | 1 | | | |
| Heterogeneity: $\text{Tau}^2 = 0.00$; $\text{Chi}^2 = 1.02$, df = 1 (P = 0.31); $I^2 = 2\%$ | | | | | | |
| Test for overall effect: Z = 0.43 (P = 0.67) | | | | | | |
| **2.1.4 Urticarial rash** | | | | | | |
| O'Doherty, B. ,1996 | 1 | 326 | 0 | 163 | 76.0% | 0.00 [-0.01, 0.01] |
| Schaad, U. B, 2002 | 1 | 141 | 0 | 130 | 24.0% | 0.01 [-0.01, 0.03] |
| **Subtotal (95% CI)** | | **467** | | **293** | **100.0%** | **0.00 [-0.01, 0.01]** |
| Total events | 2 | | 0 | | | |
| Heterogeneity: $\text{Tau}^2 = 0.00$; $\text{Chi}^2 = 0.13$, df = 1 (P = 0.72); $I^2 = 0\%$ | | | | | | |
| Test for overall effect: Z = 0.81 (P = 0.42) | | | | | | |

Risk Difference
M-H, Random, 95% CI

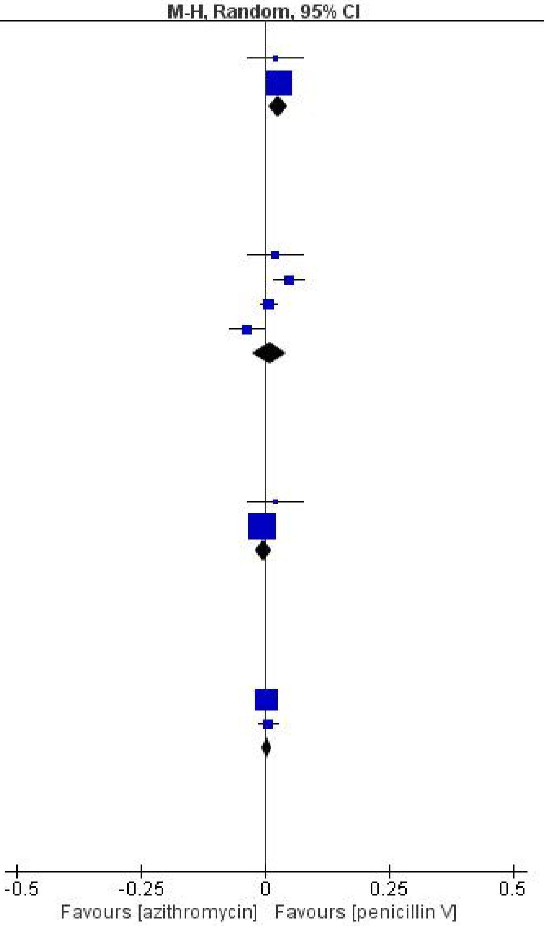


Test for subgroup differences: $\text{Chi}^2 = 6.16$, df = 3 (P = 0.10), $I^2 = 51.3\%$

## Appendix 3b: Risk difference of ADRs between azithromycin and cefaclor

| Study or Subgroup | Azithromycin Events | Azithromycin Total | Cefaclor Events | Cefaclor Total | Weight | Risk Difference M-H, Random, 95% CI |
|---|---|---|---|---|---|---|
| **3.1.1 Diarrhea** | | | | | | |
| Cremer, J., 1998 | 3 | 62 | 6 | 60 | 12.6% | -0.05 [-0.14, 0.04] |
| Oguz, F., 2003 | 1 | 41 | 1 | 37 | 21.9% | -0.00 [-0.07, 0.07] |
| Rodriguez, A. F. , 1996 | 1 | 125 | 7 | 134 | 65.4% | -0.04 [-0.09, -0.00] |
| **Subtotal (95% CI)** | | **228** | | **231** | **100.0%** | **-0.04 [-0.07, -0.00]** |
| Total events | 5 | | 14 | | | |
| Heterogeneity: Tau² = 0.00; Chi² = 1.16, df = 2 (P = 0.56); I² = 0% | | | | | | |
| Test for overall effect: Z = 2.14 (P = 0.03) | | | | | | |
| **3.1.2 Vomiting** | | | | | | |
| Cremer, J., 1998 | 1 | 62 | 2 | 60 | 62.0% | -0.02 [-0.07, 0.04] |
| Oguz, F., 2003 | 1 | 41 | 1 | 37 | 38.0% | -0.00 [-0.07, 0.07] |
| **Subtotal (95% CI)** | | **103** | | **97** | **100.0%** | **-0.01 [-0.06, 0.03]** |
| Total events | 2 | | 3 | | | |
| Heterogeneity: Tau² = 0.00; Chi² = 0.10, df = 1 (P = 0.75); I² = 0% | | | | | | |
| Test for overall effect: Z = 0.53 (P = 0.60) | | | | | | |
| **3.1.3 Abdominal pain** | | | | | | |
| Cremer, J., 1998 | 7 | 62 | 3 | 60 | 35.6% | 0.06 [-0.03, 0.16] |
| Rodriguez, A. F. , 1996 | 1 | 125 | 0 | 134 | 64.4% | 0.01 [-0.01, 0.03] |
| **Subtotal (95% CI)** | | **187** | | **194** | **100.0%** | **0.03 [-0.06, 0.11]** |
| Total events | 8 | | 3 | | | |
| Heterogeneity: Tau² = 0.00; Chi² = 3.14, df = 1 (P = 0.08); I² = 68% | | | | | | |
| Test for overall effect: Z = 0.65 (P = 0.52) | | | | | | |

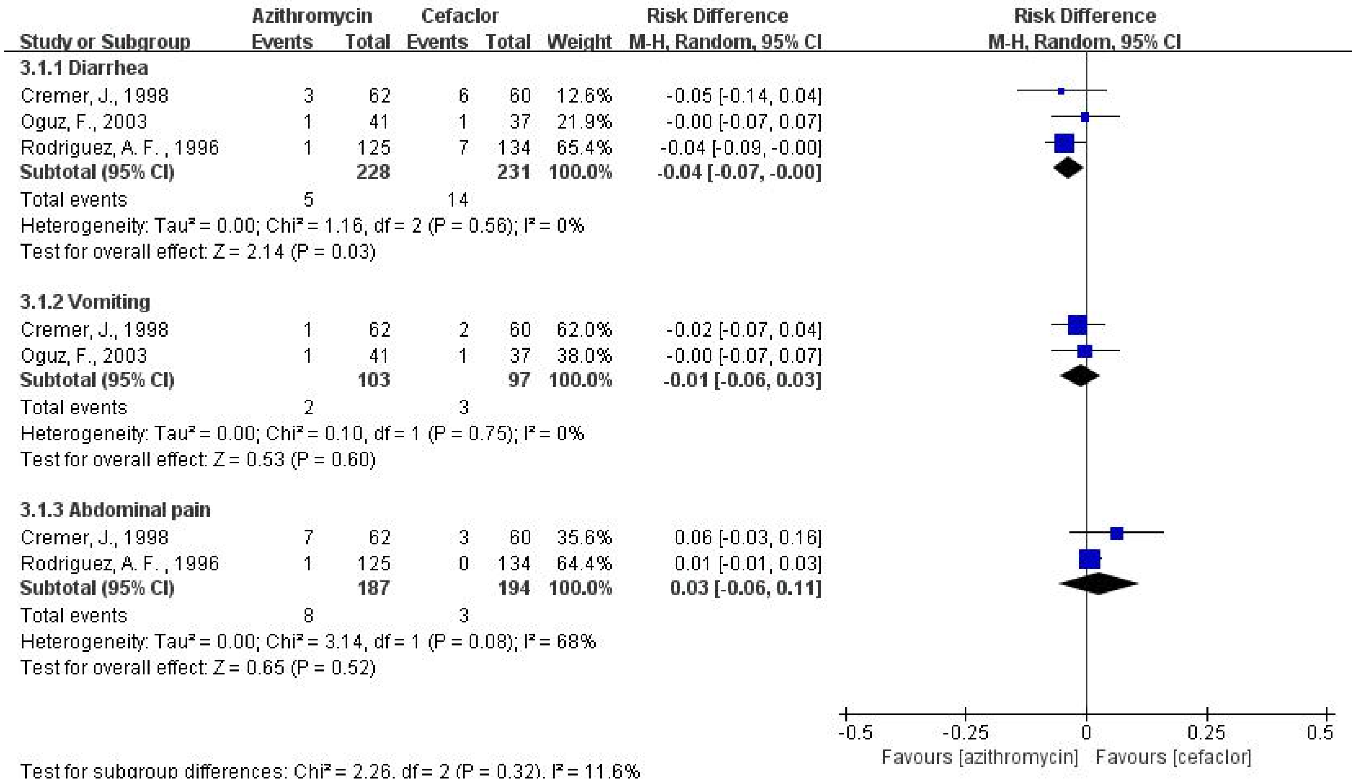


Test for subgroup differences: Chi² = 2.26, df = 2 (P = 0.32), I² = 11.6%

## Appendix 3c: Risk difference of ADRs between azithromycin and ceftriaxone

| Study or Subgroup | Azithromycin Events | Azithromycin Total | Ceftriaxone Events | Ceftriaxone Total | Weight | Risk Difference M-H, Random, 95% CI |
|---|---|---|---|---|---|---|
| **4.1.1 Diarrhea** | | | | | | |
| Arguedas, A., 2003 | 5 | 132 | 4 | 66 | 85.4% | -0.02 [-0.09, 0.04] |
| Frenck, R. W., Jr, 2004 | 10 | 32 | 15 | 36 | 7.2% | -0.10 [-0.33, 0.12] |
| Nair, B. T., 2017 | 8 | 30 | 12 | 34 | 7.3% | -0.09 [-0.31, 0.14] |
| **Subtotal (95% CI)** | | **194** | | **136** | **100.0%** | **-0.03 [-0.09, 0.03]** |
| Total events | 23 | | 31 | | | |
| Heterogeneity: Tau² = 0.00; Chi² = 1.12, df = 2 (P = 0.57); I² = 0% | | | | | | |
| Test for overall effect: Z = 1.07 (P = 0.29) | | | | | | |
| **4.1.2 Vomit** | | | | | | |
| Arguedas, A., 2003 | 3 | 132 | 0 | 66 | 54.3% | 0.02 [-0.01, 0.06] |
| Frenck, R. W., Jr, 2004 | 11 | 32 | 7 | 36 | 21.3% | 0.15 [-0.06, 0.36] |
| Nair, B. T., 2017 | 6 | 30 | 5 | 34 | 24.4% | 0.05 [-0.13, 0.24] |
| **Subtotal (95% CI)** | | **194** | | **136** | **100.0%** | **0.06 [-0.07, 0.18]** |
| Total events | 20 | | 12 | | | |
| Heterogeneity: Tau² = 0.01; Chi² = 4.60, df = 2 (P = 0.10); I² = 57% | | | | | | |
| Test for overall effect: Z = 0.92 (P = 0.36) | | | | | | |
| **4.1.3 Rash** | | | | | | |
| Arguedas, A., 2003 | 1 | 132 | 4 | 66 | 45.4% | -0.05 [-0.11, 0.01] |
| Li, D., 2005 | 0 | 23 | 3 | 22 | 18.5% | -0.14 [-0.29, 0.02] |
| Nair, B. T., 2017 | 1 | 30 | 0 | 34 | 36.2% | 0.03 [-0.05, 0.12] |
| **Subtotal (95% CI)** | | **185** | | **122** | **100.0%** | **-0.04 [-0.12, 0.04]** |
| Total events | 2 | | 7 | | | |
| Heterogeneity: Tau² = 0.00; Chi² = 4.69, df = 2 (P = 0.10); I² = 57% | | | | | | |
| Test for overall effect: Z = 0.90 (P = 0.37) | | | | | | |
| **4.1.4 Abdominal pain** | | | | | | |
| Frenck, R. W., Jr, 2004 | 5 | 32 | 5 | 36 | 47.3% | 0.02 [-0.15, 0.19] |
| Nair, B. T., 2017 | 3 | 30 | 5 | 34 | 52.7% | -0.05 [-0.21, 0.11] |
| **Subtotal (95% CI)** | | **62** | | **70** | **100.0%** | **-0.02 [-0.13, 0.10]** |
| Total events | 8 | | 10 | | | |
| Heterogeneity: Tau² = 0.00; Chi² = 0.30, df = 1 (P = 0.59); I² = 0% | | | | | | |
| Test for overall effect: Z = 0.28 (P = 0.78) | | | | | | |
| **4.1.5 Anorexia** | | | | | | |
| Frenck, R. W., Jr, 2004 | 3 | 32 | 6 | 36 | 52.8% | -0.07 [-0.23, 0.09] |
| Nair, B. T., 2017 | 3 | 30 | 6 | 34 | 47.2% | -0.08 [-0.24, 0.09] |
| **Subtotal (95% CI)** | | **62** | | **70** | **100.0%** | **-0.07 [-0.19, 0.04]** |
| Total events | 6 | | 12 | | | |
| Heterogeneity: Tau² = 0.00; Chi² = 0.00, df = 1 (P = 0.98); I² = 0% | | | | | | |
| Test for overall effect: Z = 1.27 (P = 0.20) | | | | | | |
| **4.1.6 Neutropenia** | | | | | | |
| Arguedas, A., 2003 | 19 | 132 | 3 | 66 | 21.2% | 0.10 [0.02, 0.18] |
| Frenck, R. W., Jr, 2000 | 0 | 34 | 0 | 30 | 26.0% | 0.00 [-0.06, 0.06] |
| Frenck, R. W., Jr, 2004 | 0 | 32 | 0 | 36 | 26.9% | 0.00 [-0.06, 0.06] |
| Nair, B. T., 2017 | 0 | 30 | 0 | 34 | 26.0% | 0.00 [-0.06, 0.06] |
| **Subtotal (95% CI)** | | **228** | | **166** | **100.0%** | **0.02 [-0.03, 0.08]** |
| Total events | 19 | | 3 | | | |
| Heterogeneity: Tau² = 0.00; Chi² = 9.14, df = 3 (P = 0.03); I² = 67% | | | | | | |
| Test for overall effect: Z = 0.75 (P = 0.45) | | | | | | |
| **4.1.7 Thrombocytosis** | | | | | | |
| Arguedas, A., 2003 | 0 | 132 | 0 | 66 | 74.3% | 0.00 [-0.02, 0.02] |
| Frenck, R. W., Jr, 2000 | 4 | 34 | 3 | 30 | 1.7% | 0.02 [-0.13, 0.17] |
| Frenck, R. W., Jr, 2004 | 0 | 32 | 0 | 36 | 12.7% | 0.00 [-0.06, 0.06] |
| Nair, B. T., 2017 | 0 | 30 | 0 | 34 | 11.3% | 0.00 [-0.06, 0.06] |
| **Subtotal (95% CI)** | | **228** | | **166** | **100.0%** | **0.00 [-0.02, 0.02]** |
| Total events | 4 | | 3 | | | |
| Heterogeneity: Tau² = 0.00; Chi² = 0.12, df = 3 (P = 0.99); I² = 0% | | | | | | |
| Test for overall effect: Z = 0.03 (P = 0.98) | | | | | | |

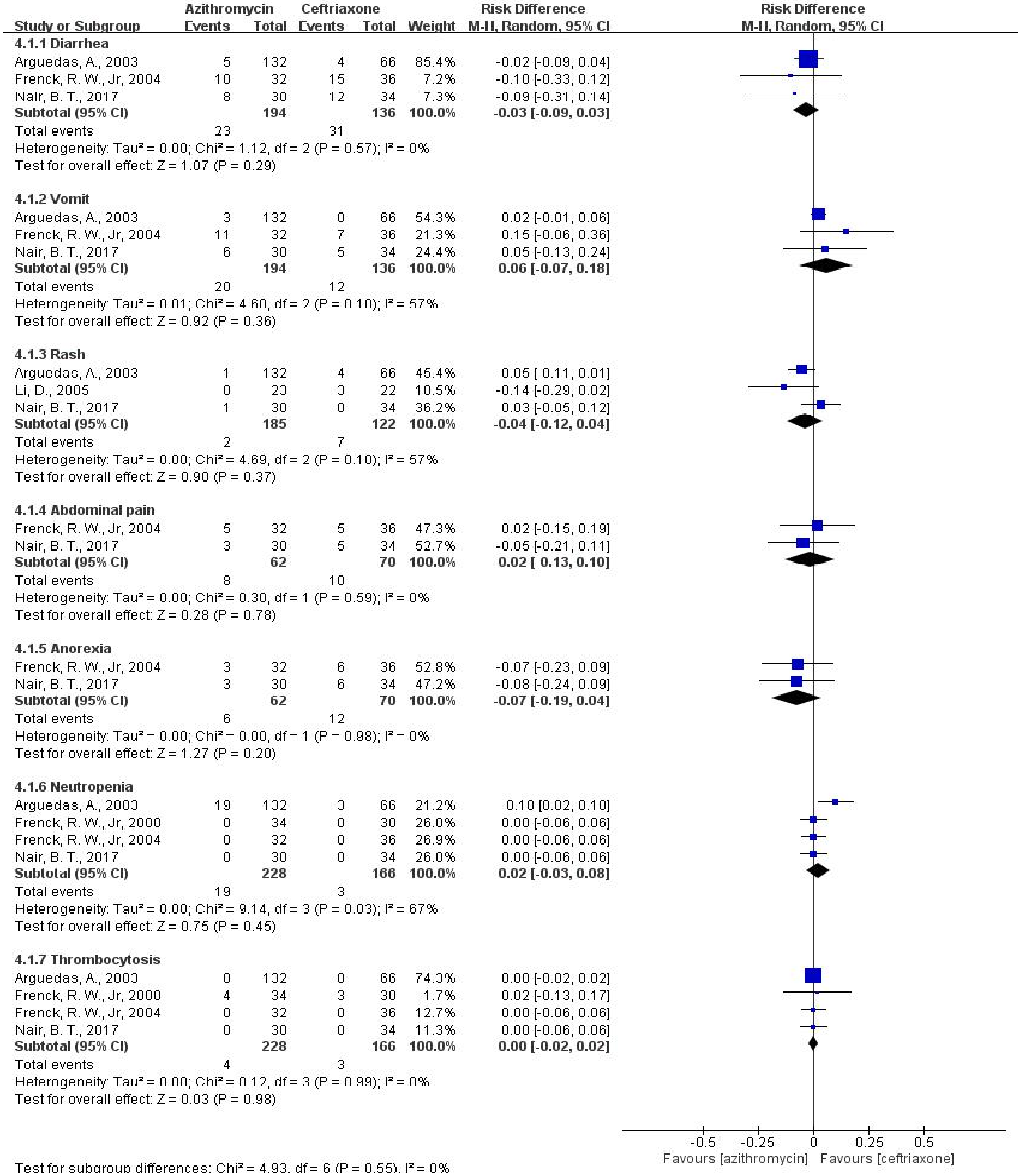


Test for subgroup differences: Chi² = 4.93, df = 6 (P = 0.55), I² = 0%

## Appendix 3d: Risk difference of ADRs between azithromycin and ceftibuten

| Study or Subgroup | Azithromycin Events | Azithromycin Total | Ceftibuten Events | Ceftibuten Total | Weight | Risk Difference M-H, Random, 95% CI |
|---|---|---|---|---|---|---|
| **5.1.1 Diarrhea** | | | | | | |
| Boccazzi, A., 2000 | 0 | 116 | 1 | 132 | 83.4% | -0.01 [-0.03, 0.01] |
| Galova,K, 1996 | 2 | 89 | 2 | 74 | 16.6% | -0.00 [-0.05, 0.04] |
| **Subtotal (95% CI)** | | **205** | | **206** | **100.0%** | **-0.01 [-0.03, 0.01]** |
| Total events | 2 | | 3 | | | |
| Heterogeneity: Tau² = 0.00; Chi² = 0.02, df = 1 (P = 0.90); I² = 0% | | | | | | |
| Test for overall effect: Z = 0.71 (P = 0.48) | | | | | | |
| **5.1.2 Eosinophilia** | | | | | | |
| Boccazzi, A., 2000 | 0 | 116 | 0 | 132 | 80.7% | 0.00 [-0.02, 0.02] |
| Galova,K, 1996 | 1 | 89 | 0 | 74 | 19.3% | 0.01 [-0.02, 0.04] |
| **Subtotal (95% CI)** | | **205** | | **206** | **100.0%** | **0.00 [-0.01, 0.02]** |
| Total events | 1 | | 0 | | | |
| Heterogeneity: Tau² = 0.00; Chi² = 0.48, df = 1 (P = 0.49); I² = 0% | | | | | | |
| Test for overall effect: Z = 0.30 (P = 0.76) | | | | | | |
| **5.1.3 Liver enzyme elevation** | | | | | | |
| Boccazzi, A., 2000 | 0 | 116 | 0 | 132 | 66.0% | 0.00 [-0.02, 0.02] |
| Galova,K, 1996 | 1 | 89 | 3 | 74 | 34.0% | -0.03 [-0.08, 0.02] |
| **Subtotal (95% CI)** | | **205** | | **206** | **100.0%** | **-0.01 [-0.05, 0.03]** |
| Total events | 1 | | 3 | | | |
| Heterogeneity: Tau² = 0.00; Chi² = 2.56, df = 1 (P = 0.11); I² = 61% | | | | | | |
| Test for overall effect: Z = 0.49 (P = 0.62) | | | | | | |

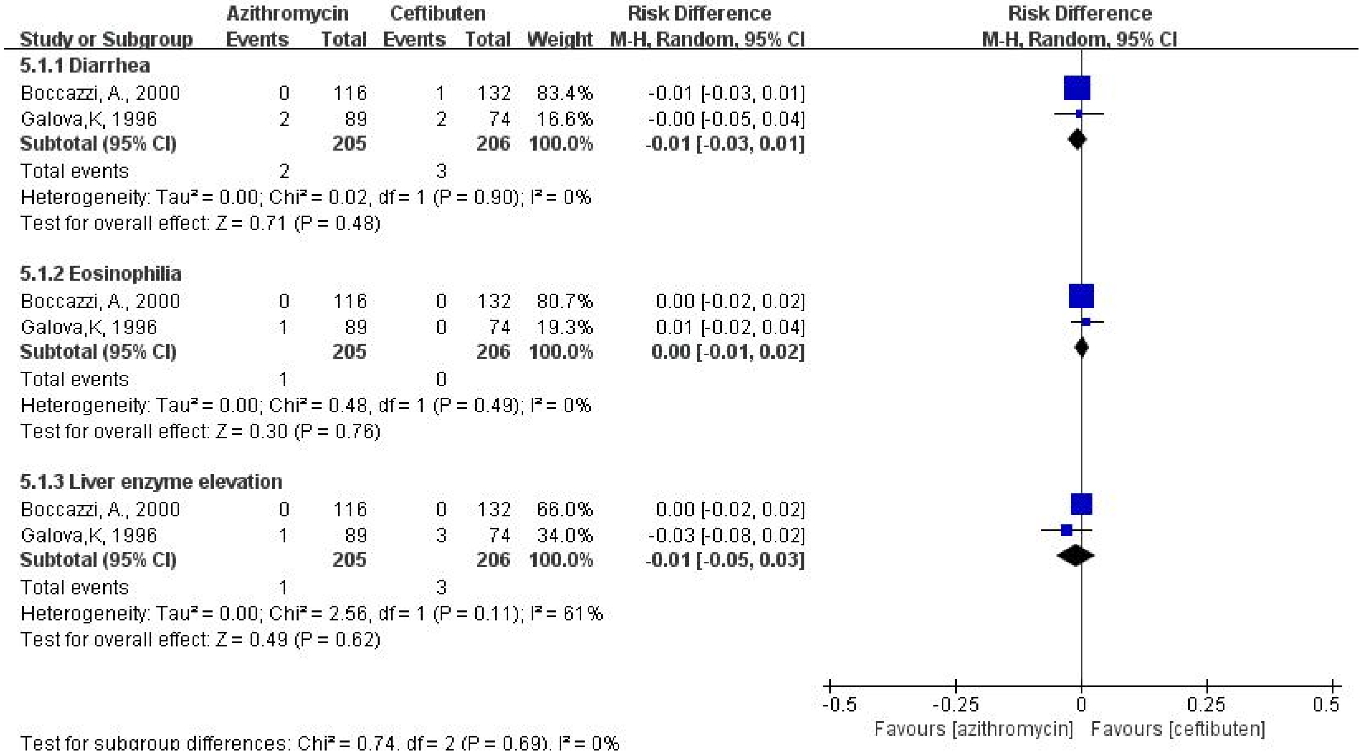


Test for subgroup differences: Chi² = 0.74, df = 2 (P = 0.69), I² = 0%

## Appendix 3e: Risk difference of ADRs between azithromycin and clarithromycin

| Study or Subgroup | Azithromycin Events | Azithromycin Total | Clarithromycin Events | Clarithromycin Total | Weight | Risk Difference M-H, Fixed, 95% CI |
|---|---|---|---|---|---|---|
| **6.1.1 Diarrhoea** | | | | | | |
| Ramet, J, 1995 | 3 | 76 | 3 | 74 | 46.3% | -0.00 [-0.06, 0.06] |
| Venuta, A.,1998 | 4 | 87 | 2 | 87 | 53.7% | 0.02 [-0.03, 0.08] |
| **Subtotal (95% CI)** | | **163** | | **161** | **100.0%** | **0.01 [-0.03, 0.05]** |
| Total events | 7 | | 5 | | | |
| Heterogeneity: Chi² = 0.33, df = 1 (P = 0.57); I² = 0% | | | | | | |
| Test for overall effect: Z = 0.57 (P = 0.57) | | | | | | |
| **Total (95% CI)** | | **163** | | **161** | **100.0%** | **0.01 [-0.03, 0.05]** |
| Total events | 7 | | 5 | | | |
| Heterogeneity: Chi² = 0.33, df = 1 (P = 0.57); I² = 0% | | | | | | |
| Test for overall effect: Z = 0.57 (P = 0.57) | | | | | | |
| Test for subgroup differences: Not applicable | | | | | | |

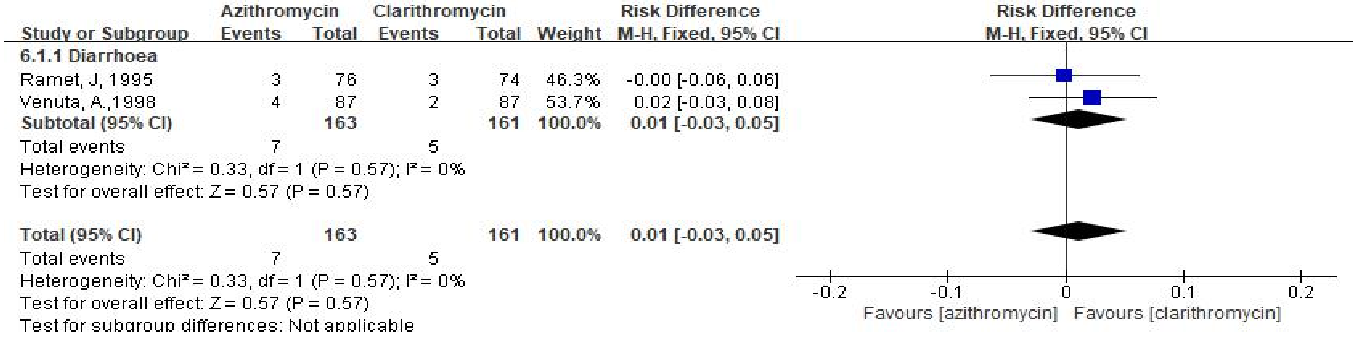

## Appendix 3f: Risk difference of ADRs between azithromycin and erythromycin

| | Azithromycin | | Erythromycin | | | Risk Difference |
|---|---|---|---|---|---|---|
| Study or Subgroup | Events | Total | Events | Total | Weight | M-H, Random, 95% CI |
| **7.1.1 Diarrhea** | | | | | | |
| Dong, 2005 | 4 | 25 | 12 | 21 | 8.2% | -0.41 [-0.67, -0.16] |
| Langley, J. M., 2004 | 17 | 239 | 28 | 238 | 37.0% | -0.05 [-0.10, 0.01] |
| Roord, J. ,1996 | 4 | 45 | 4 | 40 | 21.6% | -0.01 [-0.14, 0.11] |
| Weippl, G. , 1993 | 0 | 46 | 2 | 47 | 33.2% | -0.04 [-0.11, 0.03] |
| **Subtotal (95% CI)** | | **355** | | **346** | **100.0%** | **-0.07 [-0.15, 0.01]** |
| Total events | 25 | | 46 | | | |
| Heterogeneity: Tau² = 0.00; Chi² = 8.68, df = 3 (P = 0.03); I² = 65% | | | | | | |
| Test for overall effect: Z = 1.63 (P = 0.10) | | | | | | |
| **7.1.2 Vomiting** | | | | | | |
| Dong, 2005 | 3 | 25 | 17 | 21 | 11.0% | -0.69 [-0.90, -0.48] |
| Langley, J. M., 2004 | 12 | 239 | 31 | 238 | 19.0% | -0.08 [-0.13, -0.03] |
| Manfredi, R., 2016 | 1 | 77 | 4 | 74 | 18.8% | -0.04 [-0.10, 0.02] |
| Roord, J. ,1996 | 7 | 45 | 1 | 40 | 16.0% | 0.13 [0.01, 0.25] |
| Vukelic, D.,2010 | 0 | 60 | 1 | 30 | 17.9% | -0.03 [-0.11, 0.05] |
| Weippl, G. , 1993 | 2 | 46 | 3 | 47 | 17.3% | -0.02 [-0.11, 0.07] |
| **Subtotal (95% CI)** | | **492** | | **450** | **100.0%** | **-0.09 [-0.19, 0.02]** |
| Total events | 25 | | 57 | | | |
| Heterogeneity: Tau² = 0.01; Chi² = 48.36, df = 5 (P < 0.00001); I² = 90% | | | | | | |
| Test for overall effect: Z = 1.64 (P = 0.10) | | | | | | |
| **7.1.3 Nausea** | | | | | | |
| Langley, J. M., 2004 | 7 | 239 | 20 | 238 | 39.6% | -0.05 [-0.10, -0.01] |
| Manfredi, R., 2016 | 1 | 77 | 4 | 74 | 35.0% | -0.04 [-0.10, 0.02] |
| Roord, J. ,1996 | 4 | 45 | 0 | 40 | 25.4% | 0.09 [-0.00, 0.18] |
| **Subtotal (95% CI)** | | **361** | | **352** | **100.0%** | **-0.01 [-0.08, 0.06]** |
| Total events | 12 | | 24 | | | |
| Heterogeneity: Tau² = 0.00; Chi² = 7.86, df = 2 (P = 0.02); I² = 75% | | | | | | |
| Test for overall effect: Z = 0.38 (P = 0.70) | | | | | | |
| **7.1.4 Abdominal pain** | | | | | | |
| Dong, 2005 | 6 | 25 | 16 | 21 | 48.1% | -0.52 [-0.77, -0.27] |
| Weippl, G. , 1993 | 2 | 46 | 3 | 47 | 51.9% | -0.02 [-0.11, 0.07] |
| **Subtotal (95% CI)** | | **71** | | **68** | **100.0%** | **-0.26 [-0.85, 0.32]** |
| Total events | 8 | | 19 | | | |
| Heterogeneity: Tau² = 0.17; Chi² = 19.64, df = 1 (P < 0.00001); I² = 95% | | | | | | |
| Test for overall effect: Z = 0.88 (P = 0.38) | | | | | | |
| **7.1.5 Alanine transferase elevation** | | | | | | |
| Manfredi, R., 2016 | 0 | 77 | 0 | 74 | 54.8% | 0.00 [-0.03, 0.03] |
| Roord, J. ,1996 | 1 | 45 | 0 | 40 | 9.5% | 0.02 [-0.04, 0.08] |
| Vukelic, D.,2010 | 0 | 60 | 0 | 30 | 14.4% | 0.00 [-0.05, 0.05] |
| Weippl, G. , 1993 | 0 | 46 | 0 | 47 | 21.2% | 0.00 [-0.04, 0.04] |
| **Subtotal (95% CI)** | | **228** | | **191** | **100.0%** | **0.00 [-0.02, 0.02]** |
| Total events | 1 | | 0 | | | |
| Heterogeneity: Tau² = 0.00; Chi² = 0.52, df = 3 (P = 0.91); I² = 0% | | | | | | |
| Test for overall effect: Z = 0.22 (P = 0.83) | | | | | | |
| **7.1.6 Leukopenia** | | | | | | |
| Manfredi, R., 2016 | 0 | 77 | 0 | 74 | 52.7% | 0.00 [-0.03, 0.03] |
| Roord, J. ,1996 | 0 | 45 | 0 | 40 | 17.0% | 0.00 [-0.05, 0.05] |
| Vukelic, D.,2010 | 1 | 60 | 0 | 30 | 9.9% | 0.02 [-0.04, 0.08] |
| Weippl, G. , 1993 | 0 | 46 | 0 | 47 | 20.4% | 0.00 [-0.04, 0.04] |
| **Subtotal (95% CI)** | | **228** | | **191** | **100.0%** | **0.00 [-0.02, 0.02]** |
| Total events | 1 | | 0 | | | |
| Heterogeneity: Tau² = 0.00; Chi² = 0.31, df = 3 (P = 0.96); I² = 0% | | | | | | |
| Test for overall effect: Z = 0.17 (P = 0.86) | | | | | | |

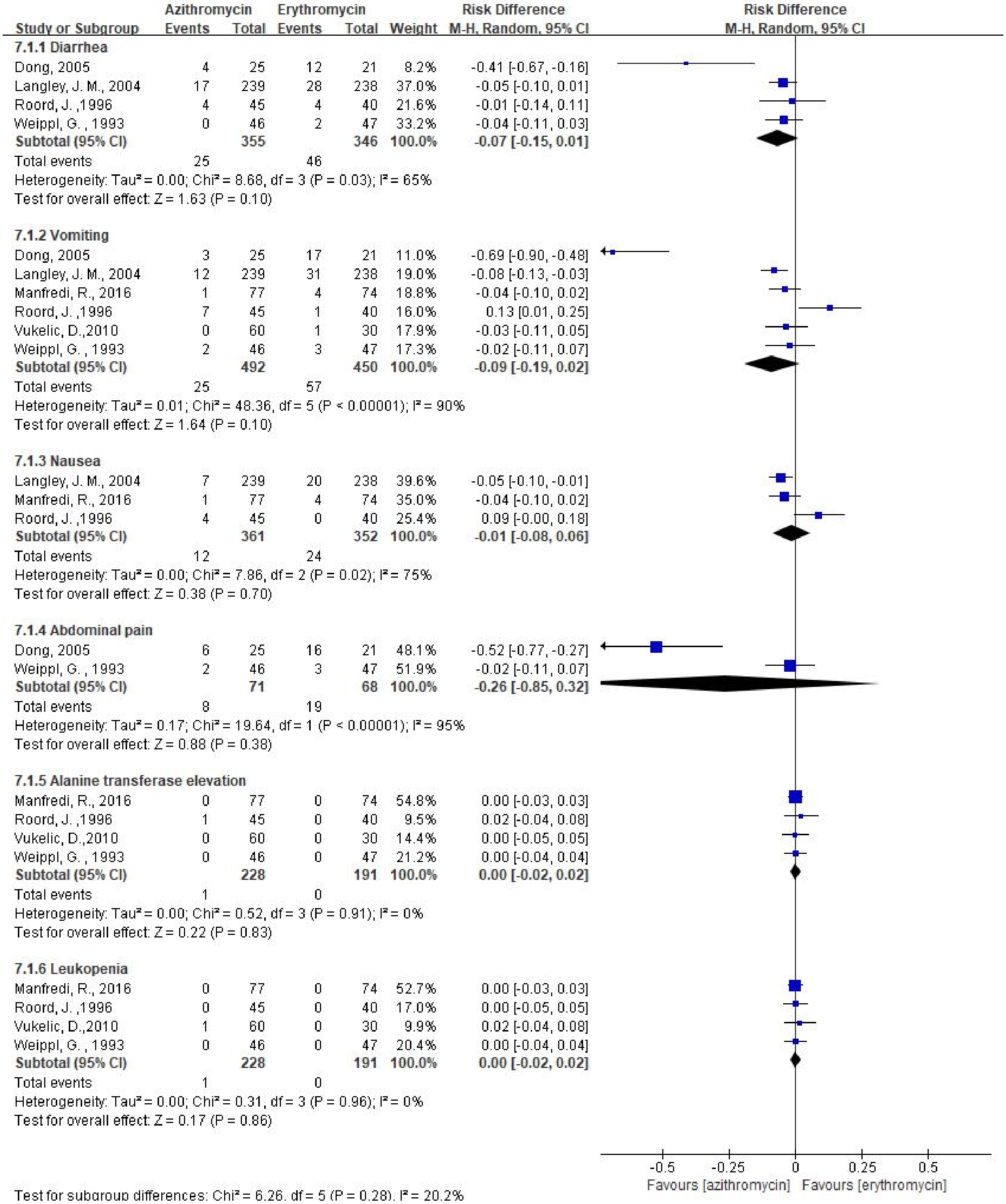


Test for subgroup differences: Chi² = 6.26, df = 5 (P = 0.28), I² = 20.2%

## Appendix 3g: Risk difference of ADRs between azithromycin and amoxicillin clavulanate

| Study or Subgroup | Azithromycin Events | Azithromycin Total | Amoxicillin clavulanate Events | Amoxicillin clavulanate Total | Weight | Risk Difference M-H, Random, 95% CI |
|---|---|---|---|---|---|---|
| **8.1.1 Diarrhea** | | | | | | |
| Arguedas, A., 2011 | 42 | 450 | 80 | 452 | 11.9% | -0.08 [-0.13, -0.04] |
| Aronovitz, G, 1996 | 1 | 85 | 15 | 84 | 8.7% | -0.17 [-0.25, -0.08] |
| Arrieta, A., 2003 | 30 | 153 | 44 | 147 | 7.8% | -0.10 [-0.20, -0.01] |
| Block, S. L., 2003 | 11 | 173 | 22 | 173 | 10.6% | -0.06 [-0.13, -0.00] |
| Ghaffar, F., 2000 | 1 | 60 | 15 | 55 | 6.3% | -0.26 [-0.38, -0.13] |
| Goyal, 2018 | 6 | 82 | 15 | 97 | 8.2% | -0.08 [-0.17, 0.01] |
| Harris, J. A, 1998 | 28 | 310 | 43 | 146 | 9.1% | -0.20 [-0.28, -0.12] |
| Hoberman, A., 2005 | 13 | 363 | 21 | 367 | 12.9% | -0.02 [-0.05, 0.01] |
| Khurana, C. M., 1996 | 3 | 263 | 25 | 263 | 12.4% | -0.08 [-0.12, -0.05] |
| McLinn, S., 1996 | 8 | 340 | 54 | 334 | 12.1% | -0.14 [-0.18, -0.10] |
| **Subtotal (95% CI)** | | **2279** | | **2118** | **100.0%** | **-0.11 [-0.15, -0.07]** |
| Total events | 143 | | 334 | | | |
| Heterogeneity: Tau² = 0.00; Chi² = 46.49, df = 9 (P < 0.00001); I² = 81% | | | | | | |
| Test for overall effect: Z = 5.24 (P < 0.00001) | | | | | | |
| **8.1.2 Vomiting** | | | | | | |
| Arguedas, A., 1996 | 4 | 47 | 6 | 45 | 3.7% | -0.05 [-0.18, 0.08] |
| Arguedas, A., 2011 | 48 | 450 | 37 | 452 | 14.3% | 0.02 [-0.01, 0.06] |
| Arrieta, A., 2003 | 8 | 153 | 12 | 147 | 10.7% | -0.03 [-0.09, 0.03] |
| Block, S. L., 2003 | 7 | 173 | 7 | 173 | 13.6% | 0.00 [-0.04, 0.04] |
| Ghaffar, F., 2000 | 2 | 60 | 1 | 55 | 10.5% | 0.02 [-0.04, 0.07] |
| Goyal, 2018 | 9 | 82 | 9 | 97 | 6.4% | 0.02 [-0.07, 0.11] |
| Harris, J. A, 1998 | 30 | 310 | 33 | 146 | 7.9% | -0.13 [-0.20, -0.05] |
| Khurana, C. M., 1996 | 5 | 263 | 8 | 263 | 16.8% | -0.01 [-0.04, 0.02] |
| McLinn, S., 1996 | 5 | 340 | 23 | 334 | 16.1% | -0.05 [-0.08, -0.02] |
| **Subtotal (95% CI)** | | **1878** | | **1712** | **100.0%** | **-0.02 [-0.05, 0.01]** |
| Total events | 118 | | 136 | | | |
| Heterogeneity: Tau² = 0.00; Chi² = 22.33, df = 8 (P = 0.004); I² = 64% | | | | | | |
| Test for overall effect: Z = 1.41 (P = 0.16) | | | | | | |
| **8.1.3 Nausea** | | | | | | |
| Arguedas, A., 1996 | 1 | 47 | 7 | 45 | 1.4% | -0.13 [-0.25, -0.02] |
| Arguedas, A., 2011 | 2 | 450 | 6 | 452 | 30.0% | -0.01 [-0.02, 0.00] |
| Block, S. L., 2003 | 3 | 173 | 2 | 173 | 17.0% | 0.01 [-0.02, 0.03] |
| Goyal, 2018 | 9 | 82 | 13 | 97 | 2.0% | -0.02 [-0.12, 0.07] |
| Harris, J. A, 1998 | 13 | 310 | 11 | 146 | 6.7% | -0.03 [-0.08, 0.01] |
| Khurana, C. M., 1996 | 3 | 263 | 5 | 263 | 20.6% | -0.01 [-0.03, 0.01] |
| McLinn, S., 1996 | 2 | 340 | 9 | 334 | 22.3% | -0.02 [-0.04, -0.00] |
| **Subtotal (95% CI)** | | **1665** | | **1510** | **100.0%** | **-0.01 [-0.03, 0.00]** |
| Total events | 33 | | 53 | | | |
| Heterogeneity: Tau² = 0.00; Chi² = 10.62, df = 6 (P = 0.10); I² = 44% | | | | | | |
| Test for overall effect: Z = 1.79 (P = 0.07) | | | | | | |
| **8.1.4 Abdominal pain** | | | | | | |
| Arguedas, A., 2011 | 11 | 450 | 7 | 452 | 19.6% | 0.01 [-0.01, 0.03] |
| Aronovitz, G, 1996 | 1 | 85 | 1 | 84 | 12.6% | -0.00 [-0.03, 0.03] |
| Arrieta, A., 2003 | 6 | 153 | 3 | 147 | 10.5% | 0.02 [-0.02, 0.06] |
| Block, S. L., 2003 | 2 | 173 | 2 | 173 | 17.3% | 0.00 [-0.02, 0.02] |
| Harris, J. A, 1998 | 15 | 310 | 18 | 146 | 5.9% | -0.07 [-0.13, -0.02] |
| Khurana, C. M., 1996 | 4 | 263 | 3 | 263 | 18.9% | 0.00 [-0.02, 0.02] |
| McLinn, S., 1996 | 7 | 340 | 15 | 334 | 15.1% | -0.02 [-0.05, 0.00] |
| **Subtotal (95% CI)** | | **1774** | | **1599** | **100.0%** | **-0.00 [-0.02, 0.01]** |
| Total events | 46 | | 49 | | | |
| Heterogeneity: Tau² = 0.00; Chi² = 14.55, df = 6 (P = 0.02); I² = 59% | | | | | | |
| Test for overall effect: Z = 0.44 (P = 0.66) | | | | | | |
| **8.1.5 Rash** | | | | | | |
| Arguedas, A., 1996 | 0 | 47 | 3 | 45 | 5.7% | -0.07 [-0.15, 0.02] |
| Arguedas, A., 2011 | 23 | 450 | 35 | 452 | 20.5% | -0.03 [-0.06, 0.01] |
| Aronovitz, G, 1996 | 0 | 85 | 2 | 84 | 16.5% | -0.02 [-0.06, 0.02] |
| Arrieta, A., 2003 | 5 | 153 | 7 | 147 | 14.3% | -0.01 [-0.06, 0.03] |
| Block, S. L., 2003 | 3 | 173 | 9 | 173 | 17.0% | -0.03 [-0.07, 0.00] |
| Goyal, 2018 | 2 | 82 | 0 | 97 | 16.5% | 0.02 [-0.01, 0.06] |
| Harris, J. A, 1998 | 19 | 310 | 18 | 146 | 9.5% | -0.06 [-0.12, -0.00] |
| **Subtotal (95% CI)** | | **1300** | | **1144** | **100.0%** | **-0.02 [-0.04, -0.00]** |
| Total events | 52 | | 74 | | | |
| Heterogeneity: Tau² = 0.00; Chi² = 9.76, df = 6 (P = 0.14); I² = 39% | | | | | | |
| Test for overall effect: Z = 2.13 (P = 0.03) | | | | | | |
| **8.1.6 Fungal dermatitis** | | | | | | |
| Arguedas, A., 2011 | 4 | 450 | 15 | 452 | 59.3% | -0.02 [-0.04, -0.01] |
| Block, S. L., 2003 | 1 | 173 | 3 | 173 | 40.7% | -0.01 [-0.03, 0.01] |
| **Subtotal (95% CI)** | | **623** | | **625** | **100.0%** | **-0.02 [-0.03, -0.00]** |
| Total events | 5 | | 18 | | | |
| Heterogeneity: Tau² = 0.00; Chi² = 0.78, df = 1 (P = 0.38); I² = 0% | | | | | | |
| Test for overall effect: Z = 2.61 (P = 0.009) | | | | | | |

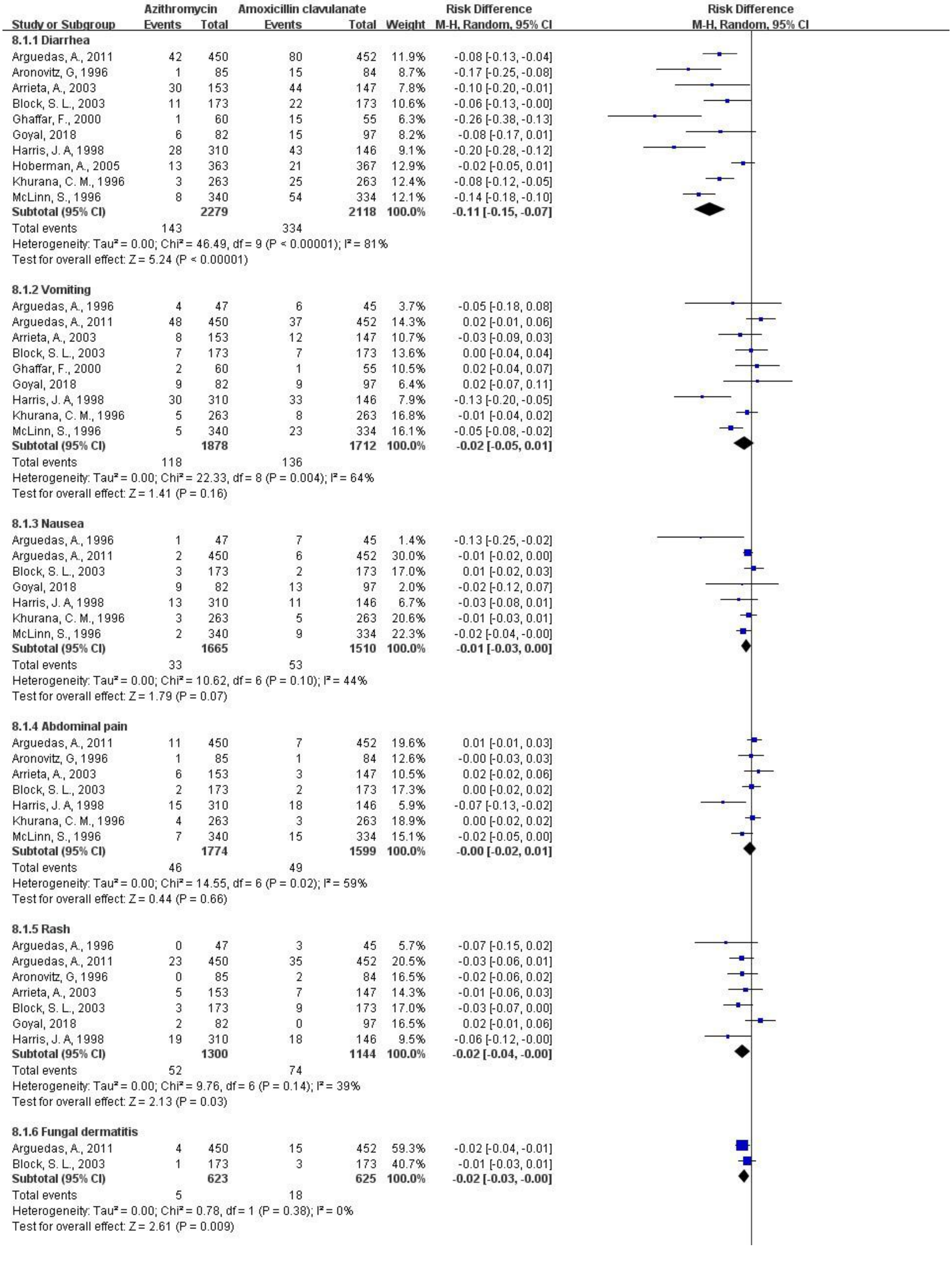

| | | | | | | |
|---|---|---|---|---|---|---|
| **8.1.7 Loose stools** | | | | | | |
| Arguedas, A., 1996 | 3 | 47 | 14 | 45 | 6.1% | -0.25 [-0.40, -0.10] |
| Arguedas, A., 2011 | 42 | 450 | 58 | 452 | 23.1% | -0.03 [-0.08, 0.01] |
| Aronovitz, G, 1996 | 1 | 85 | 9 | 84 | 16.3% | -0.10 [-0.17, -0.03] |
| Khurana, C. M., 1996 | 3 | 263 | 4 | 263 | 27.8% | -0.00 [-0.02, 0.02] |
| McLinn, S., 1996 | 6 | 340 | 14 | 334 | 26.7% | -0.02 [-0.05, 0.00] |
| **Subtotal (95% CI)** | | **1185** | | **1178** | **100.0%** | **-0.05 [-0.09, -0.00]** |
| Total events | 55 | | 99 | | | |
| Heterogeneity: Tau² = 0.00; Chi² = 22.38, df = 4 (P = 0.0002); I² = 82% | | | | | | |
| Test for overall effect: Z = 2.15 (P = 0.03) | | | | | | |
| **8.1.8 Neutropenia** | | | | | | |
| Arguedas, A., 1996 | 0 | 47 | 3 | 45 | 0.3% | -0.07 [-0.15, 0.02] |
| Arguedas, A., 2011 | 0 | 450 | 0 | 452 | 47.6% | 0.00 [-0.00, 0.00] |
| Goyal, 2018 | 0 | 82 | 0 | 97 | 3.7% | 0.00 [-0.02, 0.02] |
| Harris, J. A, 1998 | 0 | 310 | 0 | 146 | 14.2% | 0.00 [-0.01, 0.01] |
| McLinn, S., 1996 | 0 | 340 | 0 | 334 | 34.3% | 0.00 [-0.01, 0.01] |
| **Subtotal (95% CI)** | | **1229** | | **1074** | **100.0%** | **-0.00 [-0.00, 0.00]** |
| Total events | 0 | | 3 | | | |
| Heterogeneity: Tau² = 0.00; Chi² = 5.05, df = 4 (P = 0.28); I² = 21% | | | | | | |
| Test for overall effect: Z = 0.08 (P = 0.93) | | | | | | |
| **8.1.9 Abnormal liver function test** | | | | | | |
| Arguedas, A., 1996 | 4 | 47 | 5 | 45 | 0.1% | -0.03 [-0.15, 0.10] |
| Arguedas, A., 2011 | 0 | 450 | 0 | 452 | 79.7% | 0.00 [-0.00, 0.00] |
| Harris, J. A, 1998 | 0 | 310 | 0 | 146 | 13.8% | 0.00 [-0.01, 0.01] |
| McLinn, S., 1996 | 4 | 340 | 3 | 334 | 6.4% | 0.00 [-0.01, 0.02] |
| **Subtotal (95% CI)** | | **1147** | | **977** | **100.0%** | **0.00 [-0.00, 0.00]** |
| Total events | 8 | | 8 | | | |
| Heterogeneity: Tau² = 0.00; Chi² = 0.34, df = 3 (P = 0.95); I² = 0% | | | | | | |
| Test for overall effect: Z = 0.08 (P = 0.94) | | | | | | |
| **8.1.10 Fever** | | | | | | |
| Arguedas, A., 2011 | 7 | 450 | 4 | 452 | 90.4% | 0.01 [-0.01, 0.02] |
| Hoberman, A., 2005 | 37 | 363 | 38 | 367 | 9.6% | -0.00 [-0.05, 0.04] |
| **Subtotal (95% CI)** | | **813** | | **819** | **100.0%** | **0.01 [-0.01, 0.02]** |
| Total events | 44 | | 42 | | | |
| Heterogeneity: Tau² = 0.00; Chi² = 0.30, df = 1 (P = 0.58); I² = 0% | | | | | | |
| Test for overall effect: Z = 0.85 (P = 0.40) | | | | | | |
| **8.1.11 Anorexia** | | | | | | |
| Arguedas, A., 2011 | 10 | 450 | 9 | 452 | 80.9% | 0.00 [-0.02, 0.02] |
| Arrieta, A., 2003 | 5 | 153 | 4 | 147 | 19.1% | 0.01 [-0.03, 0.04] |
| **Subtotal (95% CI)** | | **603** | | **599** | **100.0%** | **0.00 [-0.01, 0.02]** |
| Total events | 15 | | 13 | | | |
| Heterogeneity: Tau² = 0.00; Chi² = 0.02, df = 1 (P = 0.88); I² = 0% | | | | | | |
| Test for overall effect: Z = 0.34 (P = 0.73) | | | | | | |
| **8.1.12 Dermatitis** | | | | | | |
| Arguedas, A., 2011 | 7 | 450 | 23 | 452 | 53.5% | -0.04 [-0.06, -0.01] |
| Arrieta, A., 2003 | 1 | 153 | 3 | 147 | 46.5% | -0.01 [-0.04, 0.01] |
| **Subtotal (95% CI)** | | **603** | | **599** | **100.0%** | **-0.03 [-0.05, -0.00]** |
| Total events | 8 | | 26 | | | |
| Heterogeneity: Tau² = 0.00; Chi² = 1.66, df = 1 (P = 0.20); I² = 40% | | | | | | |
| Test for overall effect: Z = 2.21 (P = 0.03) | | | | | | |
| **8.1.13 Thrombocytosis** | | | | | | |
| Arguedas, A., 1996 | 2 | 47 | 1 | 45 | 0.2% | 0.02 [-0.05, 0.09] |
| Arguedas, A., 2011 | 0 | 450 | 0 | 452 | 56.3% | 0.00 [-0.00, 0.00] |
| Goyal, 2018 | 0 | 82 | 0 | 97 | 2.2% | 0.00 [-0.02, 0.02] |
| Harris, J. A, 1998 | 0 | 310 | 0 | 146 | 9.8% | 0.00 [-0.01, 0.01] |
| McLinn, S., 1996 | 0 | 340 | 0 | 334 | 31.5% | 0.00 [-0.01, 0.01] |
| **Subtotal (95% CI)** | | **1229** | | **1074** | **100.0%** | **0.00 [-0.00, 0.00]** |
| Total events | 2 | | 1 | | | |
| Heterogeneity: Tau² = 0.00; Chi² = 0.53, df = 4 (P = 0.97); I² = 0% | | | | | | |
| Test for overall effect: Z = 0.02 (P = 0.98) | | | | | | |
| **8.1.14 Decreased white blood cell** | | | | | | |
| Arguedas, A., 1996 | 0 | 47 | 0 | 45 | 0.8% | 0.00 [-0.04, 0.04] |
| Arguedas, A., 2011 | 0 | 450 | 0 | 452 | 75.9% | 0.00 [-0.00, 0.00] |
| Goyal, 2018 | 0 | 82 | 0 | 97 | 3.0% | 0.00 [-0.02, 0.02] |
| Harris, J. A, 1998 | 0 | 310 | 0 | 146 | 13.1% | 0.00 [-0.01, 0.01] |
| McLinn, S., 1996 | 4 | 340 | 2 | 334 | 7.1% | 0.01 [-0.01, 0.02] |
| **Subtotal (95% CI)** | | **1229** | | **1074** | **100.0%** | **0.00 [-0.00, 0.00]** |
| Total events | 4 | | 2 | | | |
| Heterogeneity: Tau² = 0.00; Chi² = 1.07, df = 4 (P = 0.90); I² = 0% | | | | | | |
| Test for overall effect: Z = 0.21 (P = 0.83) | | | | | | |

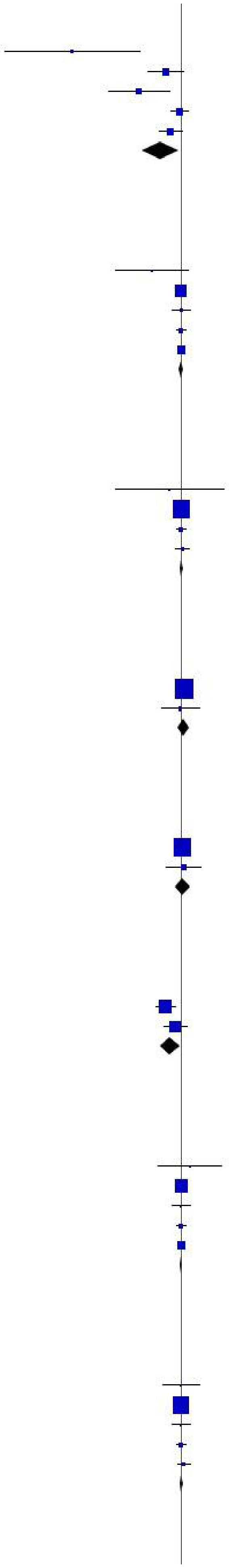

| | | | | | | |
|---|---|---|---|---|---|---|
| **8.1.15 Increased white blood cell** | | | | | | |
| Arguedas, A., 1996 | 0 | 47 | 0 | 45 | 0.8% | 0.00 [-0.04, 0.04] |
| Arguedas, A., 2011 | 0 | 450 | 0 | 452 | 71.0% | 0.00 [-0.00, 0.00] |
| Goyal, 2018 | 0 | 82 | 0 | 97 | 2.8% | 0.00 [-0.02, 0.02] |
| Harris, J. A, 1998 | 0 | 310 | 0 | 146 | 12.3% | 0.00 [-0.01, 0.01] |
| McLinn, S., 1996 | 0 | 340 | 2 | 334 | 13.1% | -0.01 [-0.02, 0.00] |
| **Subtotal (95% CI)** | | **1229** | | **1074** | **100.0%** | **-0.00 [-0.00, 0.00]** |
| Total events | 0 | | 2 | | | |
| Heterogeneity: Tau² = 0.00; Chi² = 1.47, df = 4 (P = 0.83); I² = 0% | | | | | | |
| Test for overall effect: Z = 0.42 (P = 0.67) | | | | | | |
| **8.1.16 Dedcreased red blood cell** | | | | | | |
| Arguedas, A., 1996 | 0 | 47 | 0 | 45 | 0.7% | 0.00 [-0.04, 0.04] |
| Arguedas, A., 2011 | 0 | 450 | 0 | 452 | 66.6% | 0.00 [-0.00, 0.00] |
| Goyal, 2018 | 0 | 82 | 0 | 97 | 2.6% | 0.00 [-0.02, 0.02] |
| Harris, J. A, 1998 | 0 | 310 | 0 | 146 | 11.5% | 0.00 [-0.01, 0.01] |
| McLinn, S., 1996 | 0 | 340 | 1 | 334 | 18.5% | -0.00 [-0.01, 0.01] |
| **Subtotal (95% CI)** | | **1229** | | **1074** | **100.0%** | **-0.00 [-0.00, 0.00]** |
| Total events | 0 | | 1 | | | |
| Heterogeneity: Tau² = 0.00; Chi² = 0.45, df = 4 (P = 0.98); I² = 0% | | | | | | |
| Test for overall effect: Z = 0.31 (P = 0.76) | | | | | | |
| **8.1.17 Increased eosinophils** | | | | | | |
| Arguedas, A., 1996 | 0 | 47 | 0 | 45 | 0.8% | 0.00 [-0.04, 0.04] |
| Arguedas, A., 2011 | 0 | 450 | 0 | 452 | 73.4% | 0.00 [-0.00, 0.00] |
| Goyal, 2018 | 0 | 82 | 0 | 97 | 2.9% | 0.00 [-0.02, 0.02] |
| Harris, J. A, 1998 | 0 | 310 | 0 | 146 | 12.7% | 0.00 [-0.01, 0.01] |
| McLinn, S., 1996 | 2 | 340 | 2 | 334 | 10.2% | -0.00 [-0.01, 0.01] |
| **Subtotal (95% CI)** | | **1229** | | **1074** | **100.0%** | **-0.00 [-0.00, 0.00]** |
| Total events | 2 | | 2 | | | |
| Heterogeneity: Tau² = 0.00; Chi² = 0.00, df = 4 (P = 1.00); I² = 0% | | | | | | |
| Test for overall effect: Z = 0.01 (P = 1.00) | | | | | | |
| **8.1.18 Decreased neutrophils** | | | | | | |
| Arguedas, A., 1996 | 0 | 47 | 3 | 45 | 1.6% | -0.07 [-0.15, 0.02] |
| Arguedas, A., 2011 | 0 | 450 | 0 | 452 | 39.9% | 0.00 [-0.00, 0.00] |
| Goyal, 2018 | 0 | 82 | 0 | 97 | 15.3% | 0.00 [-0.02, 0.02] |
| Harris, J. A, 1998 | 0 | 310 | 0 | 146 | 30.4% | 0.00 [-0.01, 0.01] |
| McLinn, S., 1996 | 8 | 340 | 11 | 334 | 12.7% | -0.01 [-0.03, 0.02] |
| **Subtotal (95% CI)** | | **1229** | | **1074** | **100.0%** | **-0.00 [-0.01, 0.01]** |
| Total events | 8 | | 14 | | | |
| Heterogeneity: Tau² = 0.00; Chi² = 9.90, df = 4 (P = 0.04); I² = 60% | | | | | | |
| Test for overall effect: Z = 0.42 (P = 0.67) | | | | | | |
| **8.1.19 Decreased hemoglobin** | | | | | | |
| Arguedas, A., 1996 | 0 | 47 | 0 | 45 | 0.7% | 0.00 [-0.04, 0.04] |
| Arguedas, A., 2011 | 0 | 450 | 0 | 452 | 66.6% | 0.00 [-0.00, 0.00] |
| Goyal, 2018 | 0 | 82 | 0 | 97 | 2.6% | 0.00 [-0.02, 0.02] |
| Harris, J. A, 1998 | 0 | 310 | 0 | 146 | 11.5% | 0.00 [-0.01, 0.01] |
| McLinn, S., 1996 | 0 | 340 | 1 | 334 | 18.5% | -0.00 [-0.01, 0.01] |
| **Subtotal (95% CI)** | | **1229** | | **1074** | **100.0%** | **-0.00 [-0.00, 0.00]** |
| Total events | 0 | | 1 | | | |
| Heterogeneity: Tau² = 0.00; Chi² = 0.45, df = 4 (P = 0.98); I² = 0% | | | | | | |
| Test for overall effect: Z = 0.31 (P = 0.76) | | | | | | |
| **8.1.20 Decreased glucose** | | | | | | |
| Arguedas, A., 1996 | 0 | 47 | 0 | 45 | 0.7% | 0.00 [-0.04, 0.04] |
| Arguedas, A., 2011 | 0 | 450 | 0 | 452 | 68.4% | 0.00 [-0.00, 0.00] |
| Harris, J. A, 1998 | 0 | 310 | 0 | 146 | 11.9% | 0.00 [-0.01, 0.01] |
| McLinn, S., 1996 | 0 | 340 | 1 | 334 | 19.0% | -0.00 [-0.01, 0.01] |
| **Subtotal (95% CI)** | | **1147** | | **977** | **100.0%** | **-0.00 [-0.00, 0.00]** |
| Total events | 0 | | 1 | | | |
| Heterogeneity: Tau² = 0.00; Chi² = 0.46, df = 3 (P = 0.93); I² = 0% | | | | | | |
| Test for overall effect: Z = 0.31 (P = 0.76) | | | | | | |

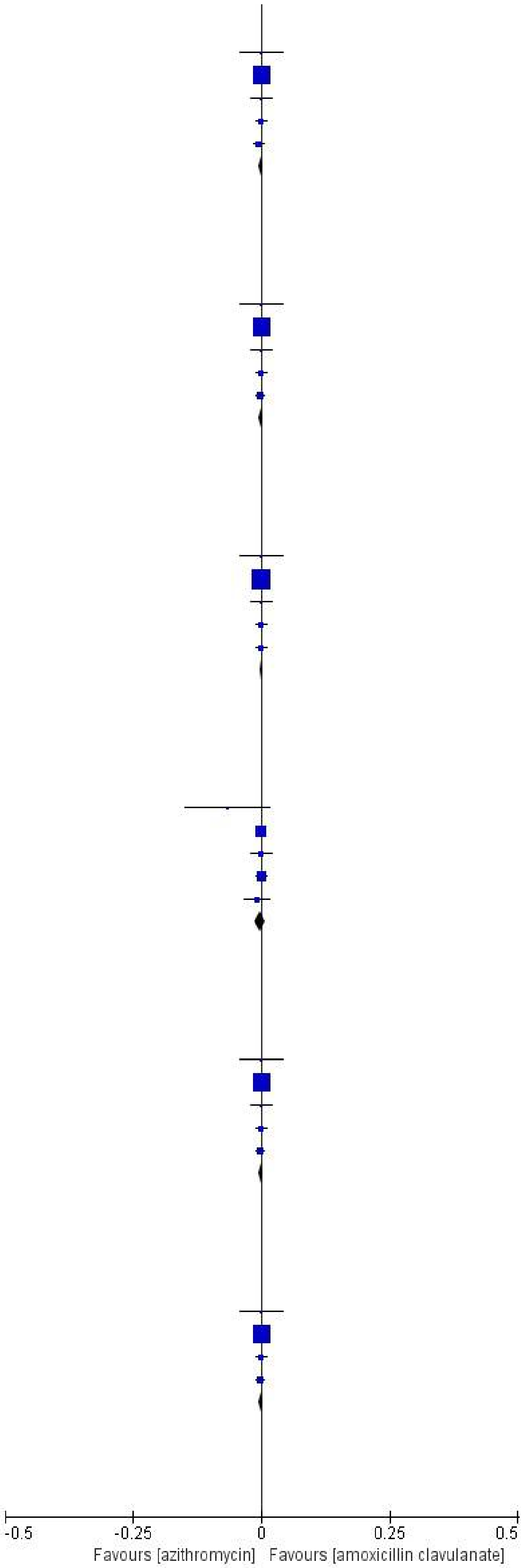


Test for subgroup differences: Chi² = 53.74, df = 19 (P < 0.0001), I² = 64.6%
