# Supplementary material for: Safety of azithromycin in pediatrics: a systematic review and meta-analysis
Source: Eur J Clin Pharmacol. 2020 Jul 17;76(12):1709–21. doi: 10.1007/s00228-020-02956-3 (PMC7661415; doi:10.1007/s00228-020-02956-3)
Supplement: Supplementary file 4 — (DOC 132 kb) [file 228_2020_2956_MOESM4_ESM.doc]

**Appendix 4 GRADE evidence profile: Azithromycin vs. placebo or other antibiotics on adverse drug reaction**s

| **Number of study (Study design)** | **Limitations**  -No serious  -Serious  -Very serious | **Inconsistency**  -No serious  -Serious  -Very serious | **Indirectness**  -No serious  -Serious  -Very serious | **Imprecision**  -No serious  -Serious  -Very serious | **Publication bias**  -Detected  -Undetected | **Certainty of evidence**  -High  -Moderate  -Low  -Very low |
| --- | --- | --- | --- | --- | --- | --- |
| **Azithromycin vs. placebo** | | | | | |  |
| **Diarrhea** | | | | | | |
| 3 RCTs | No serious | No serious | No serious | Serious | Undetected | Moderate |
| **Vomiting** | | | | | | |
| 4 RCTs | Serious | No serious | No serious | Serious | Undetected | Low |
| **Rash** | | | | | | |
| 3 RCTs | Serious | No serious | No serious | Serious | Undetected | Low |
| **Abdominal pain** | | | | | | |
| 5 RCTs | Serious | No serious | No serious | Serious | Undetected | Low |
| **Wheezing** | | | | | | |
| 3 RCTs | No serious | No serious | No serious | Serious | Undetected | Moderate |
| **Headache** | | | | | | |
| 5 RCTs | Serious | No serious | No serious | Serious | Undetected | Low |
| **Irregular heart rate** | | | | | | |
| 2 RCTs | No serious | No serious | No serious | Serious | Undetected | Moderate |
| **Chest pain** | | | | | | |
| 2 RCTs | No serious | No serious | No serious | Serious | Undetected | Moderate |
| **Constipation** | | | | | | |
| 2 RCTs | No serious | Serious | No serious | Serious | Undetected | Low |
| **Decreased appetite** | | | | | | |
| 2 RCTs | No serious | No serious | No serious | Serious | Undetected | Moderate |
| **Elevated heart rates** | | | | | | |
| 2 RCTs | No serious | No serious | No serious | Serious | Undetected | Moderate |
| **QT prolonged** | | | | | | |
| 3 RCTs | serious | No serious | No serious | Serious | Undetected | Low |
| **Cough** | | | | | | |
| 2 RCTs | No serious | Serious | No serious | Serious | Undetected | Low |
| **Neutrophil count decreased** | | | | | | |
| 3 RCTs | No serious | No serious | No serious | Serious | Undetected | Moderate |
| **Nasal congestion** | | | | | | |
| 2 RCTs | No serious | No serious | No serious | Serious | Undetected | Moderate |
| **Fever** | | | | | | |
| 3 RCTs | Serious | No serious | No serious | Serious | Undetected | Low |
| **Rhinorrhea** | | | | | | |
| 2 RCTs | Serious | No serious | No serious | Serious | Undetected | Low |
| **Productive cough** | | | | | | |
| 2 RCTs | No serious | Serious | No serious | Serious | Undetected | Low |
| **Fatigue** | | | | | | |
| 2 RCTs | No serious | No serious | No serious | Serious | Undetected | Moderate |
| **Pulmonary function decreased** | | | | | | |
| 3 RCTs | No serious | No serious | No serious | Serious | Undetected | Moderate |
| **White blood cell count decreased** | | | | | | |
| 3 RCTs | No serious | No serious | No serious | Serious | Undetected | Moderate |
| **Azithromycin vs. penicillin V** | | | | | | |
| **Diarrhea** | | | | | | |
| 2 RCTs | No serious | No serious | No serious | No serious | Undetected | High |
| **Vomit** | | | | | | |
| 4 RCTs | Serious | Serious | No serious | Serious | Undetected | Very low |
| **Headache** | | | | | | |
| 2 RCTs | Serious | No serious | No serious | Serious | Undetected | Low |
| **Urticarial rash** | | | | | | |
| 2 RCTs | No serious | No serious | No serious | Serious | Undetected | Moderate |
| **Azithromycin vs. cefaclor** | | | | | | |
| **Diarrhea** | | | | | | |
| 3 RCTs | Serious | No serious | No serious | Serious | Undetected | Low |
| **Vomiting** | | | | | | |
| 2 RCTs | Serious | No serious | No serious | Serious | Undetected | Low |
| **Abdominal pain** | | | | | | |
| 2 RCTs | Serious | No serious | No serious | Serious | Undetected | Low |
| **Azithromycin vs. ceftriaxone** | | | | | | |
| **Diarrhea** | | | | | | |
| 3 RCTs | Serious | No serious | No serious | Serious | Undetected | Low |
| **Vomit** | | | | | | |
| 3 RCTs | Serious | No serious | No serious | Serious | Undetected | Low |
| **Rash** | | | | | | |
| 3 RCTs | Serious | No serious | No serious | Serious | Undetected | Low |
| **Abdominal pain** | | | | | | |
| 2 RCTs | Serious | No serious | No serious | Serious | Undetected | Low |
| **Anorexia** | | | | | | |
| 2 RCTs | Serious | No serious | No serious | Serious | Undetected | Low |
| **Neutropenia** | | | | | | |
| 4 RCTs | Serious | No serious | No serious | Serious | Undetected | Low |
| **Thrombocytosis** | | | | | | |
| 4 RCTs | Serious | No serious | No serious | Serious | Undetected | Low |
| **Azithromycin vs. ceftibuten** | | | | | | |
| **Diarrhea** | | | | | | |
| 2 RCTs | Serious | No serious | No serious | Serious | Undetected | Low |
| **Eosinophilia** | | | | | | |
| 2 RCTs | Serious | No serious | No serious | Serious | Undetected | Low |
| **Liver enzyme elevation** | | | | | | |
| 2 RCTs | Serious | No serious | No serious | Serious | Undetected | Low |
| **Azithromycin vs. clarithromycin** | | | | | | |
| **Diarrhea** | | | | | | |
| 2 RCTs | Serious | No serious | No serious | Serious | Undetected | Low |
| **Azithromycin vs. erythromycin** | | | | | | |
| **Diarrhea** | | | | | | |
| 4 RCTs | Serious | No serious | No serious | Serious | Undetected | Low |
| **Vomiting** | | | | | | |
| 6 RCTs | Serious | Serious | No serious | Serious | Undetected | Very low |
| **Nausea** | | | | | | |
| 3 RCTs | Serious | Serious | No serious | Serious | Undetected | Very low |
| **Abdominal pain** | | | | | | |
| 2 RCTs | Serious | Serious | No serious | Serious | Undetected | Very low |
| **Alanine transferase elevation** | | | | | | |
| 4 RCTs | Serious | No serious | No serious | Serious | Undetected | Low |
| **Leukopenia** | | | | | | |
| 4 RCTs | Serious | No serious | No serious | Serious | Undetected | Low |
| **Azithromycin vs. amoxicillin clavulanate** | | | | | | |
| **Diarrhea** | | | | | | |
| 10 RCTs | Serious | Serious | No serious | No serious | Undetected | Low |
| **Vomiting** | | | | | | |
| 9 RCTs | Serious | No serious | No serious | Serious | Undetected | Low |
| **Nausea** | | | | | | |
| 7 RCTs | No serious | No serious | No serious | No serious | Undetected | High |
| **Abdominal pain** | | | | | | |
| 7 RCTs | Serious | No serious | No serious | Serious | Undetected | Low |
| **Rash** | | | | | | |
| 7 RCTs | Serious | No serious | No serious | No serious | Undetected | Moderate |
| **Fungal dermatitis** | | | | | | |
| 2 RCTs | No serious | No serious | No serious | No serious | Undetected | High |
| **Loose stools** | | | | | | |
| 5 RCTs | No serious | Serious | No serious | No serious | Undetected | Moderate |
| **Neutropenia** | | | | | | |
| 5 RCTs | No serious | No serious | No serious | No serious | Undetected | High |
| **Abnormal liver function test** | | | | | | |
| 4 RCTs | No serious | No serious | No serious | No serious | Undetected | High |
| **Fever** | | | | | | |
| 2 RCTs | No serious | No serious | No serious | Serious | Undetected | Moderate |
| **Anorexia** | | | | | | |
| 2 RCTs | No serious | No serious | No serious | Serious | Undetected | Moderate |
| **Dermatitis** | | | | | | |
| 2 RCTs | No serious | No serious | No serious | No serious | Undetected | High |
| **Thrombocytosis** | | | | | | |
| 5 RCTs | No serious | No serious | No serious | No serious | Undetected | High |
| **Decreased white blood cell** | | | | | | |
| 5 RCTs | No serious | No serious | No serious | No serious | Undetected | High |
| **Increased white blood cell** | | | | | | |
| 5 RCTs | No serious | No serious | No serious | No serious | Undetected | High |
| **Decreased red blood cell** | | | | | | |
| 5 RCTs | No serious | No serious | No serious | No serious | Undetected | High |
| **Increased eosinophils** | | | | | | |
| 5 RCTs | No serious | No serious | No serious | No serious | Undetected | High |
| **Decreased neutrophils** | | | | | | |
| 5 RCTs | No serious | No serious | No serious | Serious | Undetected | Moderate |
| **Decreased hemoglobin** | | | | | | |
| 5 RCTs | No serious | No serious | No serious | No serious | Undetected | High |
| **Decreased glucose** | | | | | | |
| 4 RCTs | No serious | No serious | No serious | No serious | Undetected | High |

Note: RCT: randomized controlled trial
