# Supplementary material for: Safety of azithromycin in pediatrics: a systematic review and meta-analysis
Source: Eur J Clin Pharmacol. 2020 Jul 17;76(12):1709–21. doi: 10.1007/s00228-020-02956-3 (PMC7661415; doi:10.1007/s00228-020-02956-3)
Supplement: Supplementary file 5 — (DOCX 21.1 kb) [file 228_2020_2956_MOESM5_ESM.docx]

**Appendix** **5 ADRs reported by retrospective studies**

| **ADRs** | **No. of events** |
| --- | --- |
| **Gastrointestinal disorders** | |
| Diarrhea | 50 |
| Vomiting | 47 |
| Abdominal pain | 27 |
| Infantile hypertrophic pyloric stenosis | 8 |
| Nausea | 8 |
| Loose stools | 2 |
| Bullous oral eruptions | 1 |
| Gastroenteritis | 1 |
| Parotid gland enlargement | 1 |
| **Subtotal** | **145** |
| **Musculoskeletal and connective tissue disorders** | |
| Tendon or joint disorders | 118 |
| **Subtotal** | **118** |
| **Investigations** | |
| Increased alanine aminotransferase increased | 7 |
| Increased aspartate aminotransferase | 4 |
| Decreased white blood cell count | 3 |
| Decrease in neutrophil count | 2 |
| Increased white blood cell counts | 2 |
| Increased gamma-glutamyl transferase | 1 |
| Liver function abnormality | 1 |
| **Subtotal** | **20** |
| **General disorders and administration site conditions** | |
| Death | 12 |
| Extravascular infiltration | 1 |
| **Subtotal** | **13** |
| **Cardiac disorders** | |
| Ventricular tachycardias | 4 |
| Cardiopulmonary resuscitation | 2 |
| Life-threatening bradyarrhythmia | 1 |
| **Subtotal** | **7** |
| **Skin and subcutaneous tissue disorders** | |
| Acute generalized exanthematous pustulosis | 1 |
| Drug reaction with eosinophilia and systemic symptoms | 1 |
| Eczema | 1 |
| Rash | 4 |
| Stevens–Johnson syndrome | 1 |
| Urticaria | 1 |
| **Subtotal** | **9** |
| **Nervous system disorders** | |
| Choreoathetosis | 1 |
| Myasthenic crisis | 1 |
| **Subtotal** | **2** |
| **Renal and urinary disorders** | |
| Acute interstitial nephritis | 1 |
| **Subtotal** | **1** |
| **Respiratory, throacic and mediastinal disorders** | |
| Asthma | 1 |
| **Subtotal** | **1** |
| **Psychiatric disorders** | |
| Agitation and choreoathetosis | 1 |
| **Subtotal** | **1** |
| **Vascular disorders** | |
| Pallor | 1 |
| **Subtotal** | **1** |
| **Immune system disorders** | |
| Hypersensitivity | **5** |
| **Subtotal** | **5** |
| **Total** | **323** |

Note: ADR: Adverse drug reaction.
